# Impacts of a Homestead Food Production Intervention on Anaemia and Micronutrient Deficiencies Among Women and Children in Rural Bangladesh: A Cluster‐Randomized Controlled Trial

**DOI:** 10.1111/mcn.70043

**Published:** 2025-05-19

**Authors:** Amanda S. Wendt, Jillian L. Waid, Anna A. Müller‐Hauser, Nathalie J. Lambrecht, Tarique Md. Nurul Huda, Nicholas N. A. Kyei, Sabine Gabrysch

**Affiliations:** ^1^ Research Department 2 Potsdam Institute for Climate Impact Research (PIK), Member of the Leibniz Association Potsdam Germany; ^2^ Heidelberg Institute of Global Health Heidelberg University Heidelberg Germany; ^3^ Helen Keller International, Bangladesh Country Office Dhaka Bangladesh; ^4^ Institute of Public Health, Charité – Universitätsmedizin Berlin, corporate member of Freie Universität Berlin and Humboldt‐Universität zu Berlin Berlin Germany; ^5^ Environmental Interventions Unit, Infectious Diseases Division icddr,b Dhaka Bangladesh; ^6^ Department of Public Health, College of Applied Medical Sciences Qassim University Buraydah Saudi Arabia

**Keywords:** haemoglobin, iron, nutrition‐sensitive agriculture, vitamin A, zinc

## Abstract

Micronutrient deficiencies affect over half of young children and two‐thirds of reproductive‐aged women worldwide. Nutrition‐sensitive agriculture interventions have the potential to increase nutrient‐dense food intake and thus improve micronutrient status. We evaluated the impact of a homestead food production (HFP) programme on micronutrient status and anaemia of women and their children (registered secondary outcomes) in the Food and Agricultural Approaches to Reducing Malnutrition (FAARM) trial, additionally assessing its impact on inflammation. We conducted a 1:1 parallel two‐arm cluster‐randomized controlled trial in Sylhet, Bangladesh, with 96 clusters. The 3‐year HFP intervention included gardening, poultry, nutrition, and hygiene components. In 2015, we conducted the baseline survey. We enrolled 2705 women and their children up to 3 years of age, and in 2019, we evaluated impacts on anaemia, iron, vitamin A, zinc and inflammation status through blood measures of these women (aged 19–44 years) and their 6‐ to 37‐month‐old children, using multilevel regression. Anaemia was common (nonpregnant women: 20%, pregnant women: 35%, children: 16%), while iron deficiency was rare among nonpregnant women (3%), 12% among pregnant women and 20% among children. Vitamin A deficiency ranged from 1% to 5%, and zinc deficiency was very common (nonpregnant women: 43%, pregnant women: 69%, children: 25%). We found no evidence of an intervention impact on micronutrient status, anaemia or inflammation among the 2483 women and 930 children measured. The moderate improvements in dietary intake achieved by the intervention were thus not sufficient, and more substantial hygiene improvements and targeted dietary changes may be needed to improve micronutrient status. This trial was registered at Clinicaltrials.gov (NCT02505711).

## Summary

- A 3‐year homestead food production intervention in Bangladesh did not improve anaemia; iron, vitamin A or zinc status; or inflammation among women and their children up to 3 years of age.
- Zinc deficiency was highly prevalent, while anaemia constituted a mild to moderate public health concern and vitamin A deficiency was a mild public health concern.
- Low iron deficiency in the region, largely due to high groundwater iron, may explain the lack of intervention impact on iron status and anaemia.
- The lack of an intervention impact on infection and inflammation may have compromised the absorption and utilization of nutrients despite improved intake.

## Graphical abstract

The Food and Agricultural Approaches to Reducing Malnutrition (FAARM) trial found no evidence that a 3‐year Homestead Food Production intervention reduced anaemia or micronutrient deficiencies among women and young children in rural Bangladesh. The intervention had improved diets and hygiene practices, but bigger changes may be needed.

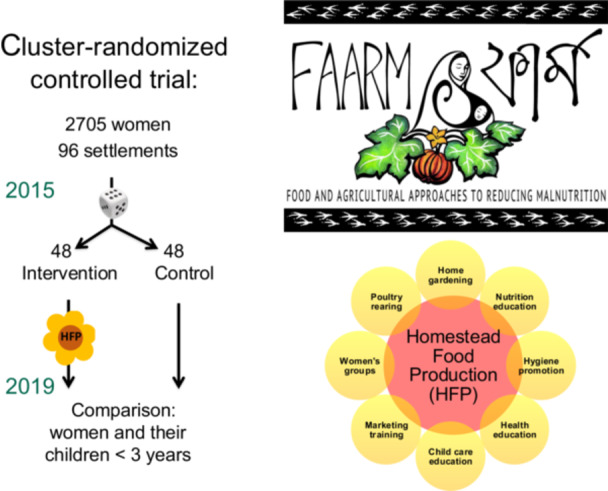

**Abbreviations**
AGP: alpha‐1‐acid glycoprotein
BRINDA: Biomarkers Reflecting Inflammation and Nutritional Determinants of Anaemia
CRP: C‐reactive protein
EDTA: dipotassium ethylenediaminetetraacetic acid
FAARM: Food and Agricultural Approaches to Malnutrition
HFP: homestead food production
LMICs: low‐ and middle‐income countries
RBP: retinol‐binding protein
SF: serum ferritin
sTfR: soluble transferrin receptor
TBI: total body iron

## 1 Introduction

Worldwide, over half of young children and two‐thirds of women of reproductive age are estimated to suffer from one or more micronutrient deficiencies (Stevens et al. 2022). These deficiencies are particularly prevalent in low‐ and middle‐income countries (LMICs), where inadequate dietary intake, poor bioavailability and a high prevalence of infections and inflammation hinder the proper absorption and utilization of nutrients (Bhutta et al. 2008). Improving diet quality in low‐income settings thus plays a crucial role in achieving Sustainable Development Goals 2.1 and 2.2, which aim to end hunger and malnutrition by 2030 (United Nations n.d.).

Micronutrient deficiencies can often manifest in subtle ways, and even mild deficiencies can lead to negative health impacts. Infants and young children are particularly vulnerable, as their nutrient needs are higher and critical for development (Kathryn 2013). Anaemia, a condition commonly associated with iron and other micronutrient deficiencies, is linked to reduced work capacity and fatigue (Haas and Brownlie 2001). For pregnant women, anaemia increases the likelihood of poor birth outcomes (e.g., low birthweight, preterm birth, neonatal mortality) and adverse maternal outcomes (e.g., postpartum haemorrhage, pre‐eclampsia, maternal death) (Young et al. 2019; Benoist et al. 2008). Iron deficiency and anaemia among children can reduce cognitive development and increase mortality risk (Jauregui‐Lobera 2014; Scott et al. 2014). Vitamin A deficiency affects various systems in the body, including vision, reproductive health and immune function (Wiseman et al. 2017), and can contribute to anaemia. Zinc plays key roles in many metabolic pathways and is, for instance, involved in immune function, growth and neurodevelopment. Zinc deficiency has been associated with poor child growth, increased child morbidity and mortality and poor maternal and birth outcomes (International Zinc Nutrition Consultative Group (IZiNCG) et al. 2004).

In rural Bangladesh, typical diets often contain large quantities of rice with small amounts of fish or vegetables. In 2018–2019 in Sylhet division, only around a quarter of people consumed a diverse diet (children: 19%, women: 27%, men: 24%), the lowest proportions in the country (BRAC James P Grant School of Public Health, National Nutrition Services 2019). Initial findings from the latest National Micronutrient Survey (2019–2020) demonstrate certain improvements in deficiencies since 2011–2012 (icddr,b, UNICEF Bangladesh, GAIN, Institute of Public Health and Nutrition 2013; Naheed 2021). However, substantial deficiencies of key micronutrients persist at levels of public health concern among women and children in Bangladesh, including iron (women: 14%, children: 15%), vitamin A (children: 7%), and in particular zinc with alarmingly high deficiency levels (women: 44%; children: 29%). These findings highlight the necessity for targeted nutritional interventions (International Zinc Nutrition Consultative Group (IZiNCG) et al. 2004; icddr,b, UNICEF Bangladesh, GAIN, Institute of Public Health and Nutrition 2013; Naheed 2021; World Health Organization 2011a, 2011b).

Nutrition‐sensitive agriculture interventions, such as homestead food production (HFP) programmes, aim to improve diets through increased access to nutrient‐dense foods, education on the importance of eating a diverse diet and improved hygiene practices (Ruel and Alderman 2013). Such interventions can also improve nutrition indirectly through increased income from surplus harvest and through women's empowerment by increasing social support and decision‐making (Ruel et al. 2018; Waid et al. 2022). While HFP programmes have shown effectiveness in improving dietary intake among women and children, their impact on anaemia has been mixed (Sharma et al. 2021; Olney et al. 2009, 2015; Osei et al. 2017; Heckert et al. 2019; Michaux et al. 2019). Additionally, research on micronutrient status beyond vitamin A is limited, and studies on inflammation or subclinical infection are lacking (Ruel et al. 2018; Sharma et al. 2021; Olney et al. 2009, 2015; Osei et al. 2017; Michaux et al. 2019). As the goal of these food‐based approaches is not to increase intake of only a single nutrient but rather to achieve a more diverse diet, which has been linked to overall increases in micronutrient adequacy (Arimond et al. 2011; Molani‐Gol et al. 2023), studies that investigate a wider range of micronutrient indicators are needed. Furthermore, although improving micronutrient status during pregnancy is often emphasized in HFP programmes, to our knowledge, no studies have assessed intervention impacts on pregnant women.

As part of the Food and Agricultural Approaches to Reducing Malnutrition (FAARM) cluster‐randomized controlled field trial, we evaluated an HFP programme's impact on anaemia; iron, vitamin A and zinc status; and inflammation among nonpregnant and pregnant women (aged 15–40 years at baseline) and their children (aged 6–37 months). Anaemia and micronutrient status (i.e., iron, vitamin A and zinc) were pre‐specified secondary trial outcomes listed in the Clinicaltrials.gov registry.

## 2 Methods

### 2.1 Study Design and Sampling

**Figure 1:**
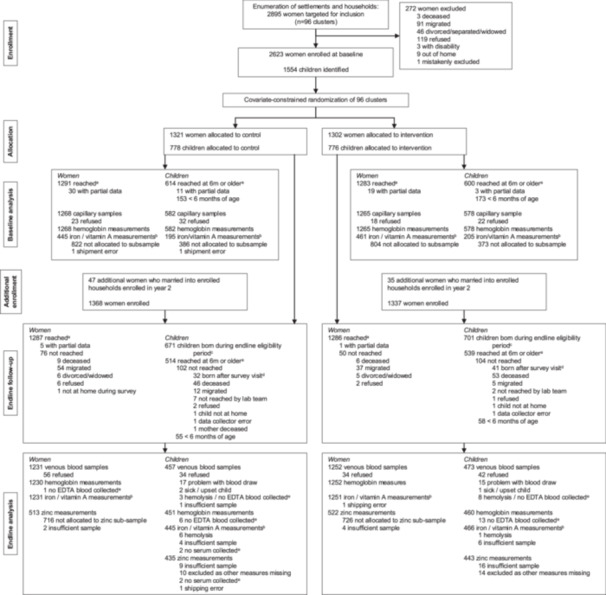
Trial profile as CONSORT flow diagram. The flowchart depicts how we arrived at the final analytic samples for the micronutrient status of women and children enrolled in the Food and Agricultural Approaches to Reducing Malnutrition (FAARM) trial in Sylhet, Bangladesh. ^a^The baseline and endline surveys consisted of several modules. The depicted numbers reflect those women and eligible children reached in the anthropometry/blood collection module (September‐December 2019). ^b^Measures included iron (serum ferritin and soluble transferrin receptor), vitamin A (retinol binding protein), and inflammation (C‐reactive protein, alpha‐1‐acid glycoprotein). ^c^The eligibility period for children was defined as those born after 1 September 2016 and before or on 22 December 2023, as all blood collection ended on this day. ^d^Later endline survey modules captured additional births that occurred after the blood collection module was complete. ^e^Blood was collected into serum and EDTA vials. In some cases, not all vials could be filled, which prevented either haemoglobin (EDTA vials) or micronutrient (serum vials) measurement. EDTA: Ethylenediaminetetraacetic acid.

The FAARM study was a two‐arm parallel cluster‐randomized trial that took place in two sub‐districts of Habiganj district in rural Sylhet, Bangladesh. The trial evaluated a 3‐year HFP intervention implemented from mid‐2015 to late‐2018. The baseline survey was conducted from March to May 2015, and the endline survey 1–1.5 years after the intervention ended (September 2019 to April 2020) to assess sustained impacts (Supporting Information S1: Figure 1). The trial was registered at Clinicaltrials.gov (NCT02505711). Impacts on other secondary outcomes evaluated in the FAARM trial have been published (Waid et al. 2024; Lambrecht et al. 2023a, 2023b; Müller‐Hauser et al. 2023). The trial included 96 settlements (clusters). After baseline, 48 were randomized to intervention and 48 to control using covariate‐constrained randomization (Lorenz and Gabrysch 2017). Women were recruited into the trial if they reported being 30 years of age or younger at enumeration in 2014; had access to at least 40 m^2^ of land, ideally with 10 m^2^ near the homestead; were married to a husband who visited the homestead at least once in the year before listing; and were interested in participating in a homestead gardening programme. We also included the recruited women's biological children aged 3 years or less, more precisely, children born after 1 March 2012 at baseline and after 1 September 2016 at endline. Thus, all endline children were exposed to the intervention from birth. The sample size was powered for the trial's primary outcome, child length‐for‐age z‐score (Wendt et al. 2019a). Randomization, inclusion criteria and blinding are detailed in Supporting Information S1: Text 1 and in the published trial protocol (Wendt et al. 2019a).

Blood measures from the baseline and endline surveys are included in the present analysis, which means we have panel data for women and data from two cross‐sectional surveys of their 6‐ to 37‐month‐old children. The subpopulation of pregnant women differed at baseline and endline (with only 29 women pregnant and providing blood measures at both time points). Further information on the sampling of subpopulations for blood measures is provided in Supporting Information S1: Text 1. At both baseline and endline, women and children with moderate/severe anaemia were referred to the local district hospital. Those with mild anaemia were informed of their condition and told to go to the nearest health facility if symptoms worsened. At baseline, this was done at point‐of‐measurement. At endline, calls were made to affected households after analysis at the field lab. More details on the study population and reasons for attrition/exclusion can be found in Figure 1.

### 2.2 Intervention Description

The objective of the FAARM trial was to evaluate the impact of a 3‐year HFP intervention, implemented by the nonprofit Helen Keller International, on child growth as the primary outcome and several secondary outcomes, including anaemia and micronutrient status. The HFP programme formed women farmer's groups and provided group training and individual counselling on year‐round gardening, poultry rearing, as well as nutrition and hygiene topics, based on the Essential Nutrition and Hygiene Actions Framework, and an additional component covering food hygiene topics (Wendt et al. 2019a; World Health Organization 2013; Sobhan et al. 2022). Group sessions and individual household visits were each conducted approximately once every 2 months, with households being contacted about once a month over the intervention time period. In the third year, households with surplus yields received marketing training and support for harvest sales. More detailed information about intervention activities is available in the published trial protocol (Wendt et al. 2019a).

We hypothesized that programme activities would lead to increased knowledge and improved practices, which would support improved access to and consumption of nutrient‐rich foods. Improved hygiene practices would additionally lead to fewer infections and reduced inflammation, improving nutrient absorption and utilization in the body (Supporting Information S1: Figure 2). Participation in HFP intervention activities was high (on average 77% group session attendance), respondents gained knowledge and adopted improved practices covered in the HFP programme, and improvements in, for example, nutrition and child feeding knowledge and garden production were documented (Waid et al. 2024; Lambrecht et al. 2023b; Sobhan et al. 2022, 2024; Kehlenbeck et al. 2024). Both intervention and control groups received certain benefits in relation to birth surveillance activities, including free pregnancy tests, birth counselling and breastfeeding counselling within 3 days of birth (Wendt et al. 2019a). Intervention spillover was not deliberately assessed; however, data on a novel garden practice (i.e., urine‐enriched biochar) showed very little spillover to control settlements in 2019 (< 1%).

### 2.3 Data Collection

Baseline survey questions covered sociodemographic characteristics, including age, religion, household wealth, maternal education, and women's pregnancy status. Wealth quintiles were calculated with principal components analysis using standard techniques (Rutstein and Johnson 2004). Pregnancy status was also assessed at the endline survey. Data sources utilized in this manuscript are outlined in Supporting Information S1:Table 1.

### 2.4 Biosample Collection

At baseline, we collected capillary blood samples, with haemoglobin measures conducted at point‐of‐contact and approximately 300 µL collected for further analyses. During the endline survey, venous blood was collected from enrolled women (8 mL) and children ≥ 183 days old (5 mL) with haematological measures conducted at the field lab on‐site (September to December 2019) and the remaining stored for further analyses. Further information on baseline and endline blood collection is detailed in Supporting Information S1: Text 2.

### 2.5 Water Collection

The population in the FAARM study area mostly consumed groundwater containing high amounts of iron. Thus, groundwater iron was measured to account for its potential effect on iron status and anaemia (Wendt et al. 2019c). From October 2019 to February 2020, water samples were collected from each of the 1443 tubewells that had been identified as an enrolled household's main source of drinking water. Groundwater iron measurements were analysed using Inductively Coupled Plasma—Optical Emission Spectroscopy (ICP‐OES) at the Institute of Earth Sciences in Heidelberg, Germany. Further details on water collection and groundwater iron analyses are described in Supporting Information S1: Text 3.

### 2.6 Laboratory Analyses

Iron status biomarkers (soluble transferrin receptor [sTfR] and serum ferritin [SF]), retinol‐binding protein (RBP) and biomarkers of inflammation (C‐reactive protein [CRP] and alpha‐1‐acid glycoprotein [AGP]) from both baseline and endline samples were analysed by the VitMin Laboratory (Willstaett, Germany) using the combined sandwich enzyme‐linked immunosorbent assay (ELISA) technique (Erhardt et al. 2004). Further quality control details can be found in Supporting Information S1: Text 4.

Zinc analyses were conducted at the University of Hohenheim Core Facility (Stuttgart, Germany) on endline samples only. The mass concentrations of serum zinc were determined using a Perkin Elmer 300x mass spectrometer with inductively coupled plasma (ICP‐MS). Details on standards and calibration procedures are detailed in Supporting Information S1: Text 4.

### 2.7 Variables

Anaemia was defined using recommended cut‐offs from the World Health Organization for nonpregnant women, pregnant women (trimester‐specific) and children (World Health Organization 2024). Anaemia definitions are detailed in Supporting Information S1: Text 5.

Iron deficiency was defined as total body iron (TBI, a measure of iron stores) below 0 mg/kg (Cook et al. 2003). We also calculated iron deficiency using SF and sTfR alone, which signal low iron stores and low iron tissue availability, respectively. Specific cut‐offs, calculations and approaches to adjust for inflammation (Namaste et al. 2017a; Rohner et al. 2017; Mei et al. 2017) are detailed in Supporting Information S1: Text 5 and the beta coefficients of the adjustment models are listed in Supporting Information S1: Table 2. Iron status unadjusted for inflammation is also reported in Supporting Information S1: Table 3.

Vitamin A status was measured using RBP, with Vitamin A deficiency defined as RBP concentration below 0.7 µmol/L, insufficiency as RBP between 0.7 and 1.05 µmol/L (for women). RBP measures for children were adjusted using internal regression correction using the Biomarkers Reflecting Inflammation and Nutritional Determinants of Anaemia (BRINDA) approach (Luo et al. 2023; Namaste et al. 2017b). Unadjusted values are listed in Supporting Information S1: Table 3 and adjustment procedures in Supporting Information S1: Table 2. For pregnant women, we used the same cut‐offs as for nonpregnant women to indicate deficiency and insufficiency; however, there is greater uncertainty in these measures, including underestimation due to lower RBP saturation during hemodilution (World Health Organization 2011b; Sapin et al. 2000).

Zinc deficiency was defined according to time of measurement and fasting status, in keeping with recommendations from the International Zinc Nutrition Consultative Group (International Zinc Nutrition Consultative Group (IZiNCG) et al. 2004). Specific cut‐offs for nonpregnant women and trimester‐specific cut‐offs for pregnant women are outlined in Supporting Information S1: Text 5. Currently, there are no universally accepted reference values for zinc deficiency among children under 3 years old. Current recommendations are to apply the cut‐off for children under 10 years old (International Zinc Nutrition Consultative Group (IZiNCG) et al. 2004). Specific cut‐offs and adjustments for inflammation (McDonald et al. 2020) are described in Supporting Information S1: Text 5. Unadjusted values are reported in Supporting Information S1: Table 3 and adjustment procedures in Supporting Information S1: Table 2.

Subclinical inflammation was determined by measures of C‐reactive protein (CRP) and alpha‐1‐acid glycoprotein (AGP), with elevated CRP defined as > 5 mg/L and elevated AGP defined as > 1 g/L. Women and children were accordingly defined as within reference values (CRP ≤ 5 mg/L and AGP ≤ 1 g/L), in incubation (only elevated CRP), early convalescence (both AGP and CRP elevated) or late convalescence (only elevated AGP) (Thurnham et al. 2010).

### 2.8 Statistical Analyses

Baseline and endline characteristics are described using frequencies and percentages for categorical variables, and arithmetic means and standard deviations for continuous variables. To compare intervention and control groups at endline, we used multilevel regression models (Stata commands *mixed* for continuous outcomes and *melogit* for binary outcomes). Impact assessments used the intention‐to‐treat approach. Assessments are presented separately for nonpregnant and pregnant women as there are different nutrient requirements and cut‐offs for deficiency during pregnancy. RBP, CRP and AGP were natural log transformed to the best fit using the Stata command *lnskew0*. All other continuous independent variables (haemoglobin, TBI, serum zinc) were not transformed as they were approximately normally distributed upon visual inspection. To reduce between‐cluster variation and thus increase power and precision, baseline individual values or cluster‐level averages of the respective outcomes were included as covariates in the models when available, as well as the month of survey and religion. Individual values were used (where available) if the woman's pregnancy status at baseline was the same as at endline; otherwise, we applied cluster‐level averages or the overall mean (for the 22 clusters without pregnant women at baseline). Models for pregnant women were additionally adjusted for the trimester, except the models for anaemia and zinc status, where there are recommended trimester‐specific cut‐offs. Model specifications with covariates and random effects are outlined in Supporting Information S1: Table 4.

We conducted sensitivity analyses to assess the influence of groundwater iron on anaemia and iron status among women and children, using both continuous and binary outcomes, to examine whether adjustment for groundwater iron changed impact estimates. For these analyses, groundwater iron values were grouped into quintiles and incorporated into the multilevel models.

All statistical analyses were conducted using Stata SE 18 (College Station, TX, USA). Impact analyses based on endline comparisons between trial arms are presented in the main text, while comparisons between endline and baseline, as well as sensitivity analyses, are presented as supplementary files.

### 2.9 Ethics Statement

The full FAARM trial protocol was positively reviewed by Heidelberg University's Medical Faculty in Germany (Reference: S‐121/2014). The trial protocol without biological sample collection was approved by the James P. Grant School of Public Health, BRAC University (Reference: 37a). Blood collection at baseline was approved by the Bangladesh Medical Research Council (Reference: BMRC/NREC/2013‐2016/844) and at endline by icddr,b (Reference: PR‐17126). Protocol changes were reported to the respective ethical boards for approval. All participants/caregivers gave written informed consent by signature or thumbprint before data collection.

## 3 Results

Of the 2705 women enrolled in the FAARM trial, 2573 were reached at the endline survey, and for 2483 women (92%), blood measures were available (haemoglobin: 2482; iron/vitamin A: 2482; zinc in a random subsample: 1035). Of the 1053 children who were reached and eligible for blood collection at endline, blood measures were available for 930 (88%) (haemoglobin: 911; iron/vitamin A: 911; zinc: 878) with similar attainment in intervention and control group (Figure 1). Attainment was similarly high at baseline when iron, vitamin A and inflammation status were measured in random sub‐samples of women and children (Figure 1). Zinc status was not measured at baseline.

Baseline characteristics are presented for women and children with blood measures at endline (Table 1) and for those with blood measures at baseline (Supporting Information S1: Table 5). One‐third of women had at least some secondary education and approximately 15% had no schooling. About two‐thirds were Muslim and one‐third were Hindu. Women who were pregnant at endline tended to be younger at baseline (Table 1). For both women and children, characteristics were similar in intervention and control groups (Table 1 and Supporting Information S1: Table 5).

**Table 1:** Baseline characteristics of women and children with any endline blood measures in the FAARM trial in Sylhet, Bangladesh.

		Nonpregnant women			Pregnant women			Endline children	
		Control	Intervention		Control	Intervention		Control	Intervention
	*n*	Freq (%)	Freq (%)	*n*	Freq (%)	Freq (%)	*n*	Freq (%)	Freq (%)
Woman's age, years	2279			204			881		
15–19		147 (12.9)	114 (10.0)		24 (25.8)	30 (27.0)		100 (23.3)	73 (16.2)
20–24		424 (37.3)	450 (39.4)		39 (41.9)	44 (39.6)		194 (45.2)	221 (48.9)
25–29		397 (34.9)	413 (36.2)		27 (29.0)	30 (27.0)		110 (25.6)	133 (29.4)
≥ 30		170 (14.9)	164 (14.4)		3 (3.2)	7 (6.3)		25 (5.8)	25 (5.5)
Child's age, months							930		
6–11		—	—		—	—		85 (18.6)	69 (14.6)
12–23		—	—		—	—		149 (32.6)	179 (37.8)
23–37		—	—		—	—		223 (48.8)	225 (47.6)
Woman's education	2279			204			881		
None		167 (14.7)	160 (14.0)		18 (19.4)	21 (18.9)		58 (13.5)	56 (12.4)
Any primary		529 (46.5)	501 (43.9)		42 (45.2)	51 (46.0)		203 (47.3)	208 (46.0)
Any secondary or higher		442 (38.8)	480 (42.1)		33 (35.5)	39 (35.1)		168 (39.2)	188 (41.6)
Household wealth quintile	2270			203			877		
First (poorest)		278 (24.5)	236 (20.8)		29 (31.5)	28 (25.2)		104 (24.2)	87 (19.4)
Second		265 (23.3)	224 (19.8)		24 (26.1)	22 (19.8)		95 (22.1)	98 (21.9)
Third		227 (20.0)	229 (20.2)		17 (18.5)	23 (20.7)		92 (21.5)	90 (20.1)
Fourth		192 (16.9)	236 (20.8)		8 (8.7)	18 (16.2)		82 (19.1)	90 (20.1)
Fifth (wealthiest)		174 (15.3)	209 (18.4)		14 (15.2)	20 (18.0)		56 (13.1)	83 (18.5)
Religion	2279			204			881		
Muslim		743 (65.3)	806 (70.6)		74 (79.6)	83 (74.8)		307 (71.6)	350 (77.4)
Hindu		395 (34.7)	335 (29.4)		19 (20.4)	28 (25.2)		122 (28.4)	102 (22.6)

*Note:* Baseline characteristics are provided for women who were/were not pregnant at endline and provided a blood measure/whose child provided a blood measure at endline. No child under age 3 at endline (2019) was also measured at baseline (2015). Characteristics are reported at the household level for the woman/child's mother and her household. Child ages were recorded when blood was taken at endline.

Abbreviation: FAARM: Food and Agricultural Approaches to Reducing Malnutrition.

Follow‐up of children enrolled in the FAARM trial stopped at around 3 years of age. Thus, the children surveyed at baseline and at endline were different as the endline survey took place over 4 years after baseline. Due to a decline in fertility of enrolled women over time and the selection of only the youngest eligible child at baseline, children with any blood measures at endline were older (mean age: 23 months) than those at baseline (mean age: 20 months), while the age distribution in intervention and control groups was largely similar at each survey (Table 1 and Supporting Information S1: Table 5). In households with children at endline, women tended to be enrolled at a younger age, but otherwise, these households were largely similar to those with children at baseline, with slight imbalances in religion between trial arms (Table 1 and Supporting Information S1: Table 5).

### 3.1 Micronutrient Status at Baseline

Around a third of both nonpregnant and pregnant women were anaemic at baseline. We found very low levels of iron deficiency among nonpregnant women (2%–5%) and pregnant women (3‐6%), which increased over the trimesters (0%–11%), depending on the iron biomarker evaluated (Supporting Information S1: Tables 6 and 7). Most women were vitamin A sufficient (nonpregnant: 71%–76%; pregnant: 60%–64%). Pregnant women had similar levels of vitamin A deficiency as nonpregnant women (nonpregnant: 3%–5%; pregnant: 3%–6%) but slightly higher levels of insufficiency (nonpregnant: 21%–24%; pregnant: 30%–37%) (Supporting Information S1: Table 5). Nearly 9 in 10 women did not have elevated inflammation biomarkers at baseline.

Around 40% of children at baseline were anaemic, and around one‐fifth were estimated to be iron deficient, as defined by total body iron (Supporting Information S1: Table 6). Twelve percent were vitamin A deficient. One‐third of children had at least one elevated inflammatory biomarker, indicating potential subclinical infection or inflammation.

### 3.2 Micronutrient Status at Endline

At endline, around one‐fifth of nonpregnant women and children and one‐third of pregnant women were anaemic, with anaemia more prevalent as pregnancies progressed (Table 2 and Supporting Information S1: Tables 6 and 8). Iron deficiency, as measured by total body iron, was very low in nonpregnant women (3%) and higher in pregnant women (12%)—particularly in the third trimester (18%)—and in children (20%). Vitamin A deficiency was low among women (nonpregnant: 1%; pregnant: 4%) and children (5%) (Supporting Information S1: Table 6). A substantial proportion of women were categorized as vitamin A insufficient, defined as RBP concentrations of 0.7–1.05 µmol/L (nonpregnant women: 15%–16%; pregnant women: 30%–37%) (Table 2). Over 40% of nonpregnant women, two‐thirds of pregnant women, and around a quarter of children were found to be zinc deficient. Most women (nonpregnant: 85%; pregnant: 82%) did not have elevated inflammatory biomarkers. As at baseline, in about one‐third of children, at least one inflammatory biomarker was elevated, with the highest percentage considered to be in late convalescence (Table 2 and Supporting Information S1: Table 6).

**Table 2:** Endline mean values and prevalence of anaemia, micronutrient deficiencies and inflammation among women and children enrolled in the FAARM trial in Sylhet, Bangladesh.

	Nonpregnant women			Pregnant women			Children		
		Control	Intervention		Control	Intervention		Control	Intervention
	*n*	*Mean (SD)* freq (%)	*Mean (SD)* freq (%)	*n*	*Mean (SD)* freq (%)	*Mean (SD)* freq (%)	*n*	*Mean (SD)* freq (%)	*Mean (SD)* freq (%)
Mean Hb (g/dL)	2278	*12.7 (0.05)*	*12.6 (0.04)*	204	*11.2 (0.09)*	*11.3 (0.12)*	911	*11.6 (0.06)*	*11.6 (0.06)*
Mean TBI (mg/kg)^a^	2279	*7.2 (0.13)*	*7.1 (0.15)*	203	*4.2 (0.40)*	*4.5 (0.43)*	911	*2.6 (0.21)*	*2.5 (0.25)*
Mean SF (µg/L)^a^	2279	*56.5 (1.31)*	*54.1 (1.53)*	203	*32.2 (3.50)*	*40.2 (3.55)*	911	*27.6 (1.10)*	*27.3 (1.24)*
Mean sTfR (mg/L)^a^	2279	*4.4 (0.05)*	*4.4 (0.05)*	203	*4.6 (0.12)*	*4.9 (0.24)*	911	*7.3 (0.13)*	*7.4 (0.18)*
Mean RBP (μmol/L)^b^	2279	*1.6 (0.03)*	*1.6 (0.03)*	203	*1.2 (0.03)*	*1.1 (0.03)*	911	*0.9 (0.01)*	*0.9 (0.01)*
Mean serum zinc (µg/L)	957	*660.0 (8.00)*	*654.2 (9.63)*	78	*467.9* (15.40)	*500.4 (19.92)*	878	*675.8 (8.49)*	*667.1 (7.30)*
Mean AGP (g/L)	2279	*0.6 (0.01)*	*0.6 (0.01)*	203	*0.4 (0.02)*	*0.4 (0.02)*	911	*0.9 (0.03)*	*0.9 (0.02)*
Mean CRP (mg/L)	2279	*2.3 (0.15)*	*2.2 (0.14)*	203	*3.1 (0.63)*	*3.3 (0.59)*	911	*2.8 (0.33)*	*3.2 (0.36)*
Anaemia^c^	2277			204			911		
None		913 (80.3)	904 (79.3)		57 (61.3)	76 (68.5)		377 (83.6)	385 (83.7)
Mild		166 (14.6)	171 (15.0)		29 (31.2)	28 (25.2)		61 (13.5)	59 (12.8)
Moderate		57 (5.0)	63 (5.5)		7 (7.5)	6 (5.4)		13 (2.9)	16 (3.5)
Severe		1 (0.1)	2 (0.2)		0 (0.0)	1 (0.9)		0 (0.0)	0 (0.0)
Iron status^a^	2279			203			911		
Deficient (TBI < 0 mg/kg)		33 (2.9)	33 (2.9)		10 (10.8)	15 (13.6)		84 (18.9)	95 (20.4)
Deficient (SF < 15 µg/L)^d^		77 (6.8)	85 (7.5)		33 (35.5)	31 (28.2)		90 (20.2)	101 (21.7)
Deficient (sTfR > 8.3 mg/L)		25 (2.2)	23 (2.0)		2 (2.2)	5 (4.6)		94 (21.1)	90 (19.3)
Vitamin A status^b^	2279			203			911		
Sufficient (RBP > 1.05 µmol/L)		936 (82.3)	954 (83.6)		59 (63.4)	66 (60.0)		418 (93.9)	446 (95.7)
Insufficient (RBP 0.7‐1.05 µmol/L)		185 (16.3)	172 (15.1)		28 (30.1)	41 (37.3)		—	—
Deficient (RBP < 0.7 µmol/L)		17 (1.5)	15 (1.3)		6 (6.5)	3 (2.7)		27 (6.1)	20 (4.3)
Zinc status^e^	957			78			878		
Deficient		197 (41.6)	217 (44.9)		27 (69.2)	27 (69.2)		114 (26.2)	104 (23.5)
Inflammatory markers^f^	2279			203			911		
Reference		965 (84.8)	972 (85.2)		78 (83.9)	89 (80.9)		306 (68.8)	328 (70.4)
Incubation		72 (6.3)	81 (7.1)		14 (15.1)	15 (13.6)		8 (1.8)	14 (3.0)
Early convalescence		46 (4.0)	36 (3.2)		0 (0.0)	1 (0.9)		51 (11.5)	53 (11.4)
Late convalescence		55 (4.8)	52 (4.6)		1 (1.1)	5 (4.6)		80 (18.0)	71 (15.2)

Abbreviations: AGP: alpha‐1‐acid glycoprotein; CRP: C‐reactive protein; FAARM: Food and Agricultural Approaches to Reducing Malnutrition; Hb: haemoglobin; RBP: retinol‐binding protein; SD: standard deviation; SF: serum ferritin; sTfR: soluble transferrin receptor; TBI: total body iron.

^a^ Iron status was adjusted for inflammation by internal regression correction (Namaste et al. 2017a; Rohner et al. 2017; Mei et al. 2017).

^b^ Vitamin A status for children was adjusted for inflammation by internal regression correction (Larson et al. 2017). Insufficient vitamin A status is not a recognized category for children, thus all children with RBP ≥ 0.7 µmol/L are listed as sufficient.

^c^ Anaemia cut‐offs: nonpregnant women ‐ mild: Hb 11–11.9 g/dL; moderate: 8.0–10.9 g/dL; severe: Hb < 8.0 g/dL; pregnant women in the first and third trimester ‐ mild: Hb 10–10.9 g/dL; moderate: 7.0–9.9 g/dL; severe: Hb < 7.0 g/dL; pregnant women in the second trimester ‐ mild: Hb 9.5–10.4 g/dL; moderate: 7.0–9.4 g/dL; severe: Hb < 7.0 g/dL; 6‐ to 23‐month‐old children ‐ mild: Hb 9.5–10.4 g/dL; moderate: 7.0–9.4 g/dL; severe: Hb < 7.0 g/dL, and ≥ 24‐month‐old children ‐ mild: Hb 10–10.9 g/dL; moderate: 7.0–9.9 g/dL; severe: Hb < 7.0 g/dL.

^d^ Different SF cut‐offs used for women (< 15 µg/L) and children (< 12 µg/L).

^e^ Zinc deficiency cut‐offs for women in the morning in a fasted state < 700 µg/L; morning, non‐fasted: < 660 µg/L; afternoon, non‐fasted: < 590 µg/L; and for children in the morning, non‐fasted: < 650 µg/L; afternoon, non‐fasted: < 570 µg/L. Zinc status for children was adjusted for inflammation by internal regression correction (International Zinc Nutrition Consultative Group (IZiNCG) et al. 2004; McDonald et al. 2020).

^f^ Inflammatory marker categories: Incubation (CRP > 5 mg/L & AGP ≤ 1 g/L), Early convalescence (CRP > 5 mg/L & AGP > 1 g/L), Late convalescence (CRP ≤ 5 mg/L & AGP > 1 g/L).

### 3.3 Trends Over Time

Between baseline (2015) and endline (2019), anaemia prevalence fell substantially among nonpregnant women (33% vs. 20%) and children (41% vs. 16%) (Supporting Information S1: Table 6). This was not the case for pregnant women, as at both time points, around one‐third of pregnant women were considered anaemic. Iron deficiency was largely similar over time among nonpregnant women but increased in pregnant women, largely due to decreases in serum ferritin. For children at endline, lower levels of serum ferritin and soluble transferrin receptor as compared to baseline led to contrasting estimates of iron deficiency as measured by serum ferritin (12% vs. 21%) and soluble transferrin receptor (33% vs. 20%), but this change did not affect total body iron measures (baseline vs. endline means: 2.9 vs. 2.6 mg/kg; iron deficiency prevalence: 18% vs. 20%). Vitamin A deficiency at endline was lower than at baseline for nonpregnant women (4% vs. 1%) and children (12% vs. 5%). Inflammation biomarkers were similar at baseline and endline for women and children.

### 3.4 Impact Assessment

Similar levels of anaemia, iron, vitamin A, zinc, and inflammation were found in the intervention and control groups at endline among both women and children (Table 2), with no evidence for impact of the HFP intervention on either the binary or continuous measures when using multilevel regression (Table 3). The uncertainty of our estimates is generally small for nonpregnant women and children, while confidence intervals are much larger for pregnant women. Intra‐cluster correlation coefficients for each outcome from null models and final models are listed in Supporting Information S1: Table 9.

**Table 3:** Impact of FAARM intervention on anaemia, micronutrient deficiencies and inflammation among women and children enrolled in the FAARM trial in Sylhet, Bangladesh.

	Nonpregnant women				Pregnant women^a^				Children^b^			
	n	*beta*/OR	95% CI	*p*‐value	n	*beta*/OR	95% CI	*p*‐value	n	*beta*/OR	95% CI	*p*‐value
Hb (g/dL)	2278	−*0.06*	(−0.14, 0.03)	0.17	204	*0.09*	(−0.19, 0.36)	0.54	911	−*0.05*	(−0.20, 0.10)	0.49
TBI (mg/kg)^c^	2279	−*0.31*	(−0.64, 0.02)	0.07	203	*0.12*	(−0.86, 1.11)	0.81	911	−*0.21*	(−0.76, 0.34)	0.46
RBP (µmol/L)^d^	2279	*0.01*	(−0.04, 0.05)	0.79	203	−*0.01*	(−0.09, 0.08)	0.91	911	−*0.006*	(−0.04, 0.03)	0.75
Serum zinc (µg/L)	957	−*14.79*	(−37.7, 8.07)	0.21	78	*16.9*	(−28.7, 62.5)	0.47	878	−*13.07*	(−34.4, 8.28)	0.23
AGP (g/L)	2279	*0.008*	(−0.04, 0.05)	0.74	203	*0.09*	(−0.07, 0.25)	0.27	911	−*0.05*	(−0.12, 0.03)	0.21
CRP (mg/L)	2279	*0.04*	(−0.16, 0.25)	0.70	203	*0.01*	(−0.34, 0.35)	0.98	911	*0.12*	(−0.17, 0.42)	0.42
Anaemia^e^	2278	1.09	(0.87, 1.37)	0.47	204	0.69	(0.36, 1.33)	0.27	911	1.09	(0.72, 1.65)	0.68
Iron deficiency (TBI < 0 mg/kg)^c^	2279	1.25	(0.71, 2.23)	0.44	203	1.40	(0.57, 3.40)	0.46	911	1.11	(0.57, 2.18)	0.76
Vitamin A deficiency (RBP < 0.7 µmol/L)^d^	2279	0.97	(0.48, 1.98)	0.94	203	0.34	(0.08, 1.51)	0.15	911	0.72	(0.40, 1.32)	0.29
Zinc deficiency^f^	957	1.43	(0.94, 2.15)	0.09	78	1.07	(0.33, 3.49)	0.91	878	0.94	(0.60, 1.45)	0.77
Inflammation (ref: below cutoffs)^g^	2279	0.98	(0.77, 1.24)	0.86	203	1.29	(0.59, 2.80)	0.52	911	0.95	(0.68, 1.32)	0.75

*Note:* Regressions for nonpregnant women and children included data from all 96 clusters. Regressions for pregnant women included data on 79 clusters for haemoglobin, iron, vitamin A, and inflammation outcomes and 53 clusters for zinc outcomes. All models adjusted for geographic clustering using random effects on the cluster level.

Abbreviations: AGP: alpha‐1‐acid glycoprotein; CI: confidence interval; CRP: C‐reactive protein; FAARM: Food and Agricultural Approaches to Reducing Malnutrition; Hb: haemoglobin; OR: odds ratio; RBP: retinol‐binding protein; TBI: total body iron.

^a^ Iron and vitamin A status regressions for pregnant women were additionally adjusted for the trimester (trimester‐specific cut‐offs were not used).

^b^ Regressions for child outcomes additionally adjusted for age as a quadratic term, child sex and included a household random effect.

^c^ Iron status was adjusted for inflammation by internal regression coefficients (Namaste et al. 2017a; Rohner et al. 2017; Mei et al. 2017).

^d^ Vitamin A status for children was adjusted for inflammation by internal regression coefficients (Larson et al. 2017).

^e^ Anaemia cut‐offs: Hb < 12.0 g/dL for nonpregnant women, Hb < 11.0 g/dL for pregnant women in the first and third trimester, Hb < 10.5 g/dL for pregnant women in the second trimester, Hb < 10.5 g/dL for 6‐ to 23‐month‐old children, and Hb < 11.0 g/dL for ≥ 24‐month‐old children.

^f^ Zinc deficiency cut‐offs for women in the morning in a fasted state < 700 µg/L; morning, non‐fasted: < 660 µg/L; afternoon, non‐fasted: < 590 µg/L; and for children in the morning, non‐fasted: < 650 µg/L; afternoon, non‐fasted: < 570 µg/L. Zinc status for children was adjusted for inflammation by internal regression correction (International Zinc Nutrition Consultative Group (IZiNCG) et al. 2004; McDonald et al. 2020).

^g^ Women and children were considered to have any inflammation if CRP > 5 mg/L or AGP > 1 g/L.

Sensitivity analyses were conducted to examine the intervention effect alongside the influence of groundwater iron on haemoglobin, anaemia and iron status, as groundwater iron is high in this region and known to influence anaemia and iron status (Wendt et al. 2019c; Rahman et al. 2016). Adjusting for groundwater iron did not alter the overall results but somewhat changed certain estimates. For example, the trend towards a negative intervention impact on total body iron among nonpregnant women was attenuated once groundwater iron was taken into account. Among nonpregnant women and children, a dose–response relationship was seen between the household‐level quintile of groundwater iron and total body iron, with a similar trend in pregnant women. (Supporting Information S1: Table 10).

## 4 Discussion

In this large‐scale cluster‐randomized controlled trial evaluating a nutrition‐sensitive agriculture programme in rural Bangladesh, there was no evidence for impact on anaemia; iron, vitamin A or zinc status; or inflammation biomarkers among women or children. This was despite improvements in dietary diversity during and post intervention (Waid et al. 2024). Anaemia and vitamin A status improved among nonpregnant women and children from baseline to endline, regardless of trial assignment. In contrast, iron deficiency increased among pregnant women over time.

Our results do not support any beneficial effect of the FAARM trial's HFP intervention on haemoglobin or anaemia among nonpregnant women and children (the upper limits of the confidence intervals are ≤ 0.1 g/dl), while the evidence is less clear among the much smaller sample of pregnant women where the findings are still compatible with a 0.3 g/dl increase in haemoglobin. Previous studies have reported mixed results, and prior nutrition‐sensitive agriculture interventions that did not include a nutrition‐specific component, such as micronutrient powders, often failed to impact anaemia (Olney et al. 2009, 2015; Osei et al. 2017; Heckert et al. 2019; Michaux et al. 2019; Angeles‐Agdeppa et al. 2019; Le Port et al. 2017; Erismann et al. 2017; Osei et al. 2015; Passarelli et al. 2020). However, three earlier cluster‐randomized controlled trials of Helen Keller International's HFP programme in Burkina Faso, Cambodia, and Nepal did find anaemia reductions among children and, in Nepal, also among women (Olney et al. 2015; Osei et al. 2017; Michaux et al. 2019). The trial in Burkina Faso found a reduction in anaemia among a sub‐sample of children (3–5.9 months old at baseline) in one of the two intervention arms, which delivered behaviour change messages through health committee members (vs. through older women leaders), but no evidence of an effect in the full target group (Olney et al. 2015). Baseline anaemia levels among enrolled children in Burkina Faso were much higher than in the FAARM population (~90% vs. ~40% anaemic). The trial in Cambodia also found an improvement in anaemia among children 6–59 months old in one intervention arm, which conducted an enhanced HFP programme (vs. the same programme with the addition of fish ponds) (Michaux et al. 2019). They reported similar increases in haemoglobin across the two intervention arms (0.25 g/dL) in comparison to a control arm. Baseline child anaemia prevalence in this study was also somewhat higher than in the FAARM population (~60%). The HFP trial in Nepal found haemoglobin improvements among both children (12–48 months old) and women (Osei et al. 2017). Baseline anaemia prevalence in Nepal was similar to the FAARM enrolled women and lower than our enrolled children, potentially due to children in the Nepal trial being slightly older (mean age: 29 months vs. 20 months in FAARM) (Braat et al. 2024).

The FAARM trial's lack of impact on haemoglobin and anaemia may partly be due to alternative etiologies of anaemia in our study population (e.g., thalassaemia (Wendt et al. 2024)). Studies at the FAARM site and in the region found that some groundwater sources contain high iron concentrations, which has been associated with anaemia and iron status (Wendt et al. 2019c; Rahman et al. 2016; Wendt et al. 2019b; Merrill et al. 2011). Thus, our intervention's modest increase in iron‐rich food intake, largely through plant‐based sources with lower bioavailability, may not have meaningfully changed iron status in the context of relatively high groundwater iron intake (Waid et al. 2024). Sensitivity analyses showed groundwater iron was a stronger predictor of anaemia and iron status than the intervention (Supporting Information S1: Table 10).

In both the intervention and control group, anaemia decreased over time among nonpregnant women and children. Part of this reflects national improvements reported among children in the Bangladesh National Micronutrient Surveys (icddr,b, UNICEF Bangladesh, GAIN, Institute of Public Health and Nutrition 2013; Naheed 2021). Seasonality might also play a role, with the endline survey starting around 1 month after Eid al‐Adha, a period of high meat consumption, while the baseline survey occurred before the Boro rice harvest, a typically lean period. Differences in blood sampling methods (capillary at baseline, venous at endline) also complicate comparisons over time. Previous studies comparing venous and capillary blood for haemoglobin measures have found slightly different estimates, both higher and lower, translating to differing anaemia prevalence (Neufeld et al. 2019). Recent work recommends pooled capillary blood (mixing several drops of finger‐pricked blood with anticoagulant), or venous blood, over single drops of capillary blood, which is more typically done (De la Cruz‐Góngora et al. 2022). During our endline survey, we also measured haemoglobin using capillary blood in a sub‐sample to explore the role of the blood matrix. We found overall higher haemoglobin estimates with venous blood, leading to lower anaemia prevalence (Wendt et al. 2020), suggesting that this may explain part of the anaemia reduction. Anaemia estimates for pregnant women, however, did not substantially change between the baseline and endline survey. The pregnant women sub‐samples at the two time points did differ in composition, i.e., age, and it is conceivable that factors like wealth and education influence fertility and family planning choices over time and are thus at least partly responsible for this discrepancy.

We also found no evidence for impact of the HFP intervention on iron deficiency among women and children. This was not surprising given the low prevalence of iron deficiency, especially among nonpregnant women. As mentioned above, the lack of impact is likely due to the fact that much of the iron intake in this population is through groundwater. To our knowledge, only one other trial of a nutrition‐sensitive agriculture intervention has examined iron status and found no change in iron status among a sub‐sample of women of reproductive age (Michaux et al. 2019).

No intervention impact was found for vitamin A deficiency, as measured through RBP concentrations. Our results can nevertheless not exclude substantial reductions or increases in deficiency in both women and children, given the wide confidence intervals. Similar to iron, we also found relatively little vitamin A deficiency among women. Baseline prevalence among children was 12%, lower than other studies that found an impact (29%–73%) (Low et al. 2007; Hotz et al. 2012), but still constituting a moderate public health problem (World Health Organization 2011b). The generally low levels of deficiency in the population may have affected our ability to detect a change, both statistically and biologically, as RBP is less sensitive to dietary intakes due to homoeostatic regulation in the presence of adequate liver stores (Olson 1984).

This lack of impact was despite increased intake of vitamin A‐rich foods among women and children, though the increase was < 10% more women/children eating these foods (Waid et al. 2024). Increases were also largely in plant‐based foods, which contain less bioavailable vitamin A, with small increases (~6%) in egg consumption. Previous nutrition‐sensitive agricultural interventions that found an impact on inadequate vitamin A status largely targeted the promotion of one vitamin A‐rich crop, the orange‐fleshed sweet potato (Low et al. 2007; Hotz et al. 2012; Girard et al. 2017). FAARM's HFP programme did distribute orange flesh‐sweet potato cuttings but as part of a diverse basket of promoted crops. This suggests that to influence vitamin A status, an increased intake of foods that supply substantial vitamin A may be needed. An enhanced HFP programme combined with an aquaculture intervention in Cambodia, however, did improve the continuous RBP measure among women but not children (Michaux et al. 2019). This was also reflected in dietary intake estimates in that study, which showed increased vitamin A intake among only women (Verbowski et al. 2018).

Our results also provide no evidence for an intervention impact on zinc deficiency for women or children, but there is again substantial uncertainty around the estimates, especially for children and pregnant women, which means we cannot rule out effects in either direction. The HFP intervention successfully increased and improved poultry ownership, which led to a modest increase in egg intake (Lambrecht et al. 2023b) but not poultry or meat consumption (Waid et al. 2024). As a nutrient required for general metabolism, zinc is tightly regulated, and dietary deficiencies result in a rapid reduction of endogenous loss, followed by generalized consequences, such as growth faltering and wasting (Golden 1989). Plasma zinc is a useful population‐level indicator of zinc deficiency and is commonly used in nutritional surveys. However, it is not very sensitive to short‐term zinc inputs from food and is affected by other factors (e.g., infections and severe stress) (King et al. 2015). Although our intervention aimed to improve zinc consumption over a long period of time, the modest increase in egg intake was likely not enough to meaningfully improve zinc status. Only one other trial evaluated zinc status in an HFP programme. They also found no change among women and did not assess children (Michaux et al. 2019). Our zinc deficiency estimates are in line with recent national prevalence estimates, with over 40% of women and a third of children classified as zinc deficient (Naheed 2021), more than twice the cut‐off (> 20%) to indicate a public health problem.

Despite delivering hygiene messages and a concentrated food hygiene curriculum (Sobhan et al. 2022), we did not see improvements in the inflammation biomarkers CPR and AGP. While we primarily measured these as correction factors for iron, inflammation is also of interest in and of itself as it influences nutrient absorption. Similar to CRP and AGP, we did not see an intervention impact on other indicators such as food contamination, diarrhoea, acute respiratory infection or environmental enteric dysfunction (Lambrecht et al. 2023a; Müller‐Hauser et al. 2023; Huda et al. 2025). While the intervention positively affected food hygiene behaviours, they were not consistently practiced, and hand‐washing remained low overall (Sobhan et al. 2024; Huda et al. 2025), which may explain the lack of impact. One could speculate that if we had been more successful in reducing inflammation and subclinical intestinal infections through improved hygiene practices (Müller‐Hauser et al. 2023), we may have seen improvements in micronutrient status as women and children may have been more able to absorb and utilize the additional nutrients consumed.

### 4.1 Strengths and Limitations

As a large cluster‐randomized‐controlled trial evaluating a 3‐year nutrition‐sensitive agriculture intervention, this study has a strong design and long‐term time horizon. Children born to enrolled women during the trial were exposed to the intervention from preconception and throughout gestation, potentially benefitting for the full 1000 days. We included multiple biomarkers of iron status (i.e., serum ferritin and soluble transferrin receptor) and adjusted iron, vitamin A and zinc status for inflammation according to the current recommended standards (Mei et al. 2017; McDonald et al. 2020; Larson et al. 2017). For iron, vitamin A and inflammation, but not zinc, we additionally collected biomarker data at baseline, enabling us to adjust for baseline values in the impact assessment. As clusters were randomly allocated and arms were well balanced, this was not necessary but was done, as prespecified, to increase precision.

Limitations include the low prevalence of iron or vitamin A deficiency among women—which limited our ability to detect improvements. Another limitation is that we only collected blood during the baseline and endline surveys, and do not have any biomarker data for the interim years. The surveys also occurred in different seasons, which makes the temporal comparisons more difficult to interpret. The endline survey, conducted 1–1.5 years post‐intervention, may have missed peak intervention effects as dietary improvements waned over time (Waid et al. 2024). We purposely assessed post‐intervention impacts as many intervention evaluations are only conducted immediately after the intervention ends, while ours sought to ascertain whether sustained impacts were achieved. However, unlike other outcomes that we assessed (e.g., dietary diversity, poultry ownership), micronutrient values were not measured in between during surveillance activities; thus, we have no information on what, if any, changes appeared during the time of highest dietary impact. Multiple rounds of blood collection during or shortly after the intervention ended would have provided more information, especially on pregnant women, and could have increased the sample size of this critical group to detect potential changes at different pregnancy trimesters. However, we were not able to carry this out due to the financial and logistical constraints involved with establishing a field lab, and the trial was not designed to detect differences among pregnant women. Despite analysing a range of nutritional biomarkers, we may have missed others that improved due to intervention activities. Planned analyses will assess micronutrient intake quantitatively, potentially identifying promising indicators for future assessment in stored blood samples. It is also possible that more substantial improvements may not yet manifest in the first 3 years of children's lives and that later follow‐up studies may be needed to detect delayed impacts.

As part of their 2019 National Food and Nutrition Security Policy, the Bangladesh government has acknowledged the importance of ‘nutrition‐sensitive diversification’, including HFP systems as well as nutrition messaging, which emphasizes locally available food‐based strategies (Ministry of Food, Government of Bangladesh 2019). In 2021, they initiated a *Pushti Bagan* (nutrition garden) project with the aim of creating gardens on unused land nationwide (Agriculture Minister 2021). Increasing access to and consumption of nutrient‐dense foods is a critical step in the right direction to alleviate the burden of malnutrition in vulnerable households and communities. From our experience, we recommend that future evaluations of such programmes in any setting take into account baseline micronutrient levels, alternative nutrient sources (e.g., groundwater iron), and consider other practices that may inhibit or enhance the absorption of nutrients consumed through an improved diet (e.g., hygiene and food safety). Given the high levels of zinc deficiency among women and children in our study population, additional micronutrient supplementation may be needed to effectively address this critical deficiency.

## 5 Conclusions

Overall, we did not see an impact of the evaluated HFP intervention on anaemia; iron, vitamin A or zinc status; or inflammation among women or children. As iron deficiency in the study region was found to be low, largely due to the high iron levels in the groundwater, little change in iron status was expected. However, the lack of impact on anaemia and other micronutrients measured (i.e., vitamin A and zinc) may indicate that the moderate improvements in dietary intake observed (Waid et al. 2024) were too small to make a difference. As the intervention also did not reduce infection (Lambrecht et al. 2023a) and inflammation, this may have also affected respondents' ability to absorb and utilize the nutrients consumed, resulting in little change in micronutrient status.

## Author Contributions

A.S.W., J.L.W. and S.G. conceived of the study and analyses. S.G. is the principal investigator of the FAARM trial. A.S.W., J.L.W. and S.G. oversaw baseline and endline data collection. A.A.M., N.N.A.K., T.M.N.H. supervised and oversaw endline blood collection and analysis. J.L.W. processed the data. A.S.W. conducted analyses and drafted the manuscript. J.L.W., A.A.M., N.J.L., T.M.N.H., N.N.A.K., and S.G. gave critical feedback and revised manuscript drafts. All authors read and approved the final manuscript.

## Conflicts of Interest

The authors declare no conflicts of interest.

## Supporting information

Supporting information Figure 1**: Timeline of FAARM intervention and survey activities.** The graph depicts years and months from 2014 to 2019, the implementation of the intervention (in yellow), its proposed outputs (in green), the survey recall periods (in blue) and trajectories of the oldest and youngest children that could have had blood measures taken (6‐37 months) during baseline and endline surveys (in orange). The Food and Agricultural Approaches to Reducing Malnutrition (FAARM) trial was conducted in Habiganj District, Sylhet, Division, Bangladesh, from mid‐2015 to late 2018 (full intervention in dark yellow, roll‐out and scale‐down in light yellow). The intervention aimed to improve traditional Homestead Food Production (HFP) and reached its optimum implementation in the last year (dark green). During the baseline survey (March‐May 2015), we collected capillary blood samples from 2533 women and 1160 children. During the endline survey, venous blood was collected from 2483 women and 930 children. Month abbreviations: January (J), March (M), May (M), July (J), September (S), November (N). **Supplemental Figure 2**: **Hypothesized theory of change.** This graph spells out how the Food and Agricultural Approaches to Reducing Malnutrition (FAARM) intervention may have impacted micronutrient status among women and children in Sylhet, Bangladesh, based on the pre‐specified impact paths as outlined in the FAARM protocol paper (Wendt et al. 2019a).

Supporting information_tables_final.

Supporting information_texts_final.

## Data Availability

A deidentified data set with the individual participant data that underlie the results reported in this article is available to interested researchers who provide a methodologically sound proposal for use of the data. Data requests with a proposal should be directed to the corresponding author (ASW; amanda.wendt@pik-potsdam.de) and the principal investigator (SG; sabine.gabrysch@charite.de). A data access agreement will need to be signed to gain access to the data. The FAARM trial protocol is available online.
