# Supplementary material for: Impacts of a Homestead Food Production Intervention on Anaemia and Micronutrient Deficiencies Among Women and Children in Rural Bangladesh: A Cluster‐Randomized Controlled Trial
Source: Matern Child Nutr. 2025 May 19;21(4):e70043. doi: 10.1111/mcn.70043 (PMC12454213; doi:10.1111/mcn.70043)
Supplement: Supplementary file 1 — Supporting information Figure 1: Timeline of FAARM intervention and survey activities. The graph depicts years and months from 2014 to 2019, the implementation of the intervention (in yellow), its proposed outputs (in green), the survey recall periods (in blue) and trajectories of the oldest and youngest children that could have had blood measures taken (6‐37 months) during baseline and endline surveys (in orange). The Food and Agricultural Approaches to Reducing Malnutrition (FAARM) trial was conducted in Habiganj District, Sylhet, Division, Bangladesh, from mid‐2015 to late 2018 (full intervention in dark yellow, roll‐out and scale‐down in light yellow). The intervention aimed to improve traditional Homestead Food Production (HFP) and reached its optimum implementation in the last year (dark green). During the baseline survey (March‐May 2015), we collected capillary blood samples from 2533 women and 1160 children. During the endline survey, venous blood was collected from 2483 women and 930 children. Month abbreviations: January (J), March (M), May (M), July (J), September (S), November (N). Supplemental Figure 2: Hypothesized theory of change. This graph spells out how the Food and Agricultural Approaches to Reducing Malnutrition (FAARM) intervention may have impacted micronutrient status among women and children in Sylhet, Bangladesh, based on the pre‐specified impact paths as outlined in the FAARM protocol paper (Wendt et al. 2019a). [file MCN-21-e70043-s002.pdf]

Supplemental figures

- **Supplemental Figure 1**: Timeline of FAARM intervention and survey activities
- **Supplemental Figure 2**: Hypothesized theory of change

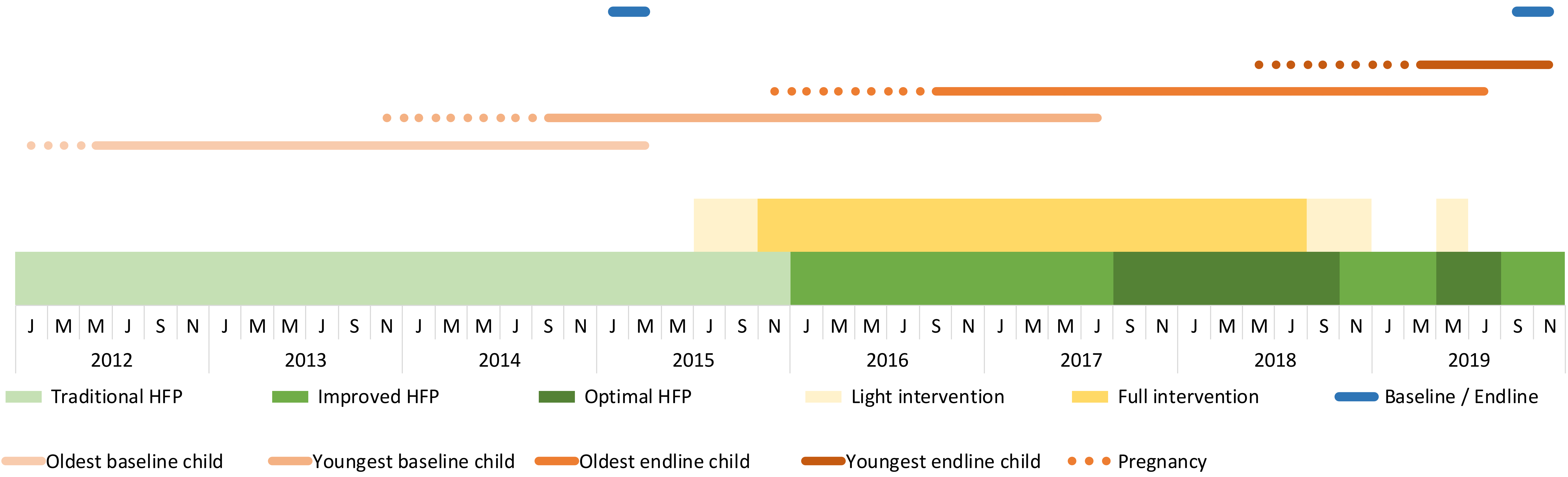

Supplemental Figure 1: Timeline of FAARM intervention and survey activities

The graph depicts years and months from 2014 to 2019, the implementation of the intervention (in yellow), its proposed outputs (in green), the survey recall periods (in blue) and trajectories of the oldest and youngest children that could have had blood measures taken (6-37 months) during baseline and endline surveys (in orange).The Food and Agricultural Approaches to Reducing Malnutrition (FAARM) trial was conducted in Habiganj District, Sylhet, Division, Bangladesh, from mid-2015 to late 2018 (full intervention in dark yellow, roll-out and scale-down in light yellow). The intervention aimed to improve traditional Homestead Food Production (HFP) and reached its optimum implementation in the last year (dark green).

During the baseline survey (March-May 2015), we collected capillary blood samples from 2533 women and 1160 children. During the endline survey, venous blood was collected from 2483 women and 930 children. Month abbreviations: January (J), March (M), May (M), July (J), September (S), November (N).

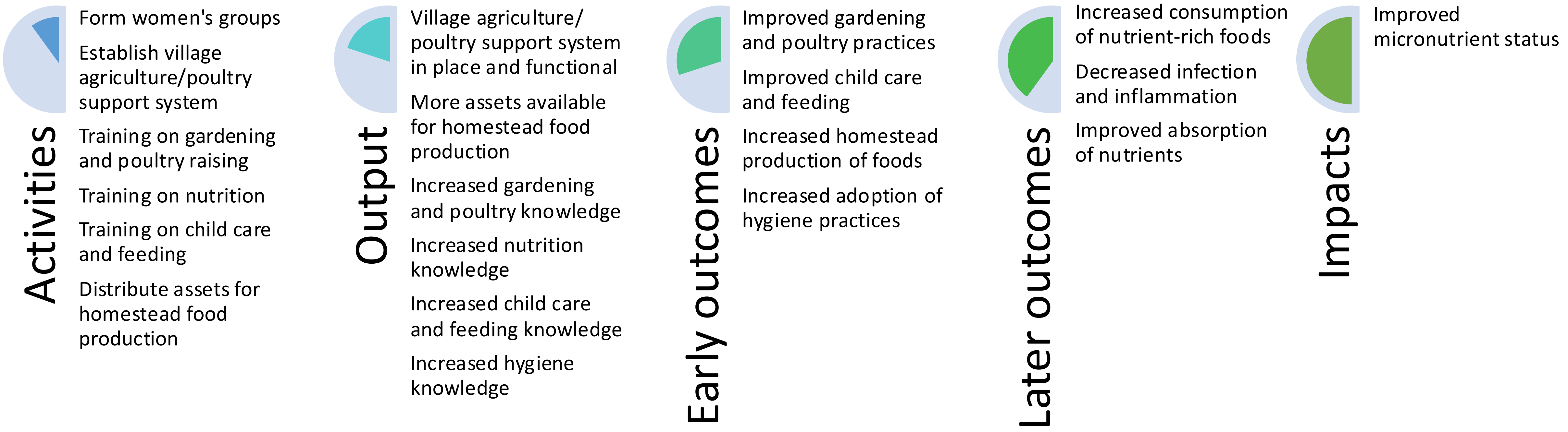

Supplemental Figure 2: Hypothesized theory of change

This depicts how the Food and Agricultural Approaches to Reducing Malnutrition (FAARM) intervention may have impacted on micronutrient status among women and children in Sylhet, Bangladesh, based on the pre-specified impact paths as outlined in the FAARM protocol paper [34].
