# Supplementary material for: Impacts of a Homestead Food Production Intervention on Anaemia and Micronutrient Deficiencies Among Women and Children in Rural Bangladesh: A Cluster‐Randomized Controlled Trial
Source: Matern Child Nutr. 2025 May 19;21(4):e70043. doi: 10.1111/mcn.70043 (PMC12454213; doi:10.1111/mcn.70043)
Supplement: Supplementary file 2 — Supporting information_tables_final. [file MCN-21-e70043-s001.pdf]

Supplemental tables

- Supplemental Table 1: Summary of FAARM data collection sources supporting this manuscript
- Supplemental Table 2: Beta coefficients and percentiles used to calculate inflammation adjustments
- Supplemental Table 3: Unadjusted values for iron and vitamin A status among women and children enrolled in the FAARM trial in Sylhet, Bangladesh
- Supplemental Table 4: Model specifications including covariate adjustment and random effects
- Supplemental Table 5: Baseline characteristics of women and children with any baseline blood measures in the FAARM trial in Sylhet, Bangladesh
- Supplemental Table 6: Baseline and endline mean values and prevalence of anemia, micronutrient deficiencies, and inflammation among women and children enrolled in the FAARM trial in Sylhet, Bangladesh
- Supplemental Table 7: Baseline biological parameters by trimester among pregnant women in the FAARM trial in Sylhet, Bangladesh
- Supplemental Table 8: Endline biological parameters by trimester among pregnant women in the FAARM trial in Sylhet, Bangladesh
- Supplemental Table 9: Intra-cluster correlation coefficients and cluster sizes for anemia, micronutrient deficiencies, and inflammation among women and children enrolled in the FAARM trial in Sylhet, Bangladesh
- Supplemental Table 10: Effect of the intervention and of groundwater iron levels on anemia and iron status among women and children enrolled in the FAARM trial in Sylhet, Bangladesh

Supplemental Table 1: Summary of FAARM data collection sources supporting this manuscript

Data collection system	Data collection time period	Blood collection	Type of other data provided and where used	Data collector characteristics	Sample targeted
Baseline survey	March - May 2015	Yes	Individual and household characteristics (Table 1 & Supp Table 4); Pregnancy trimester (Supp Table 6 & 7)	Short-term contracted	All enrolled women (survey concurrent with enrollment)
Baseline survey - new women's enrollment	October 2016 - December 2016	No	Individual characteristics (Table 1 & Supp Table 4)	Short-term contracted	Women who married into intervention households
Program data	June 2015 - May 2019	No	Attendance (Main text; Methods)	Field facilitators (implementers)	Intervention group women only
Endline survey - Water collection module	October 2019 - February 2020	No	Groundwater iron (Supp Table 10)	Full-time, field-site based & short-term contracted	Households of all enrolled women
Endline survey - Blood collection module	October 2019 - December 2019	Yes	Pregnancy trimester (Supp Table 6, 7 & 9)	Short-term contracted	All enrolled women

GAAP2: Second phase of the Gender, Agriculture, and Assets Project
FAARM: Food and Agricultural Approaches to Reducing Malnutrition
COVID: Coronavirus disease 2019

Supplemental Table 2: Beta coefficients and percentiles used to calculate inflammation adjustments

	Beta coefficients		10th percentile			
	lnCRP	lnAGP	lnCRP	CRP	lnAGP	AGP
Non-pregnant women			-3.689	0.025	-1.079	0.340
SF	0.070	0.019				
sTfR		0.197				
Pregnant women			-1.802	0.165	-1.470	0.230
SF	-0.062	0.855				
sTfR		0.016				
Children			-3.689	0.025	-0.844	0.430
SF	0.034	0.351				
sTfR		0.128				
RBP	-0.051	-0.012				
Serum zinc	-11.336	0.589				

SF: serum ferritin; sTfR: soluble transferrin receptor; RBP: retinol-binding protein

MB: micronutrient biomarker; obs: observed values; ref: reference value, defined as the lowest decile (10th percentile) of CRP / AGP; $\boldsymbol{\beta}_1$: CRP regression coefficient; $\boldsymbol{\beta}_2$: AGP regression coefficient

Table values were used for the following formula: $\ln MB_{adjusted} = \ln MB_{unadjusted} - \boldsymbol{\beta}_1(\ln CRP_{obs} - \ln CRP_{ref}) - \boldsymbol{\beta}_2(\ln AGP_{obs} - \ln APG_{ref})$ which was then back-transformed to obtain $MB_{adjusted}$. Adjustment was applied when $CRP_{obs} > CRP_{ref}$, $AGP_{obs} > AGP_{ref}$, or both

In cases where MB was only adjusted for AGP, the following equation was used: $\ln MB_{adjusted} = \ln MB_{unadjusted} - \boldsymbol{\beta}_2(\ln AGP_{obs} - \ln APG_{ref})$ which was then back-transformed to obtain $MB_{adjusted}$. Adjustment was applied when $AGP_{obs} > AGP_{ref}$

Supplemental Table 3: Unadjusted values for iron and vitamin A status among women and children enrolled in the FAARM trial in Sylhet, Bangladesh

	Non-pregnant women						Pregnant women						Children					
	Baseline			Endline			Baseline			Endline			Baseline			Endline		
	n	% or mean	(95% CI)	n	% or mean	(95% CI)	n	% or mean	(95% CI)	n	% or mean	(95% CI)	n	% or mean	(95% CI)	n	% or mean	(95% CI)
Mean TBI (mg/kg)	773	7.4	(7.1, 7.7)	2279	7.6	(7.4, 7.8)	133	7.0	(6.4, 7.6)	203	5.4	(4.8, 6.0)	400	3.4	(3.0, 3.7)	911	3.4	(3.1, 3.7)
Mean SF (μg/L)	773	67.9	(64.6, 71.2)	2279	71.3	(68.5, 74.1)	133	60.5	(53.6, 67.4)	203	45.2	(38.5, 51.8)	400	39.4	(36.6, 42.2)	911	38.3	(35.9, 40.7)
Mean sTfR (mg/L)	773	5.2	(5.0, 5.4)	2279	4.9	(4.8, 4.9)	133	5.2	(4.5, 5.9)	203	4.8	(4.5, 5.1)	400	9.0	(8.5, 9.6)	911	7.9	(7.7, 8.2)
Mean RBP (μmol/L)[a]	773	1.4	(1.4, 1.5)	2279	1.6	(1.6, 1.7)	133	1.2	(1.1, 1.2)	203	1.2	(1.1, 1.2)	400	0.9	(0.8, 0.9)	911	0.9	(0.9, 0.9)
Mean serum zinc (μg/L)[a]	-	-	-	957	657.1	(644.7, 669.5)	-	-	-	78	484.2	(458.9, 509.4)	-	-	-	878	671.4	(660.4, 682.5)
Iron deficiency																		
Deficient (TBI < 0 mg/kg)	773	1.8	(0.7, 2.9)	2279	2.5	(1.8, 3.3)	133	3.0	(0.2, 5.9)	203	9.4	(5.3, 13.4)	400	15	(11.3, 18.7)	911	15.9	(13.0, 18.8)
Deficient (SF < 15 μg/L)[b]	773	3.2	(1.7, 4.7)	2279	5.0	(3.8, 6.1)	133	5.3	(1.5, 9.0)	203	25.6	(19.0, 32.2)	400	9.0	(5.8, 12.2)	911	14.2	(11.6, 16.7)
Deficient (sTfR < 8.3 mg/L)	773	4.0	(2.5, 5.5)	2279	2.9	(2.2, 3.6)	133	4.5	(1.1, 7.9)	203	3.4	(1.0, 5.9)	400	37.8	(33.1, 42.4)	911	29.0	(25.5, 32.4)
Vitamin A deficiency (RBP < 0.7 μmol/L)	773	4.0	(2.5, 5.5)	2279	1.4	(0.9, 1.9)	133	4.5	(0.5, 8.5)	203	4.4	(1.7, 7.2)	400	25	(20.3, 29.7)	911	18.9	(16.3, 21.5)
Zinc deficiency[c]	-	-	-	957	43.3	(38.8, 47.7)	-	-	-	78	69.2	(58.2, 80.2)	-	-	-	878	34.9	(31.3, 38.4)

FAARM: Food and Agricultural Approaches to Reducing Malnutrition; CI: confidence interval; TBI: total body iron; SF: serum ferritin; sTfR: soluble transferrin receptor; RBP: retinol-binding protein

[a] RBP and serum zinc were not adjusted for inflammation among women, as recommended by the BRINDA approach

[b]Different SF cut-offs used for women (< 15 μg/L) and children (< 12 μg/L)

[c]Zinc deficiency cut-offs for women in the morning in a fasted state < 700 μg/L; morning, non-fasted: < 660 μg/L; afternoon, non-fasted: < 590 μg/L; and for children in the morning, non-fasted: < 650 μg/L; afternoon, non-fasted: < 570 μg/L.

Supplemental Table 4: Model specifications including covariate adjustment and random effects

Population group	Outcome	Variable type	Stata command	Covariates	Random effects
Non-pregnant women	Hemoglobin (g/dL)	Continuous	mixed	Baseline hemoglobin, month of survey, religion	Settlement
Non-pregnant women	Total body iron (mg/kg)[a]	Continuous	mixed	Baseline total body iron value, month of survey, religion	Settlement
Non-pregnant women	Retinol-binding protein (µmol/L)[b]	Continuous	mixed	Baseline retinol-binding protein, month of survey, religion	Settlement
Non-pregnant women	Serum zinc (µg/L)	Continuous	mixed	Month of survey, religion	Settlement
Non-pregnant women	Mean alpha-1-acid glycoprotein (g/L)	Continuous	mixed	Baseline alpha-1-acid glycoprotein, month of survey, religion	Settlement
Non-pregnant women	Mean C-reactive protein (mg/L)	Continuous	mixed	Baseline C-reactive protein, month of survey, religion	Settlement
Non-pregnant women	Anemia[c]	Binary	melogit	Baseline hemoglobin, month of survey, religion	Settlement
Non-pregnant women	Iron deficiency (TBI < 0 mg/kg)[a]	Binary	melogit	Baseline total body iron value, month of survey, religion	Settlement
Non-pregnant women	Vitamin A deficiency (RBP < 0.7 µmol/L)[b]	Binary	melogit	Baseline retinol-binding protein, month of survey, religion	Settlement
Non-pregnant women	Zinc deficiency[d]	Binary	melogit	Month of survey, religion	Settlement
Non-pregnant women	Inflammation (ref: Healthy)[e]	Binary	melogit	Baseline alpha-1-acid glycoprotein and C-reactive protein values, month of survey, religion	Settlement
Pregnant women	Hemoglobin (g/dL)	Continuous	mixed	Baseline hemoglobin, trimester, month of survey, religion	Settlement
Pregnant women	Total body iron (mg/kg)[a]	Continuous	mixed	Baseline total body iron value, trimester, month of survey, religion	Settlement
Pregnant women	Retinol-binding protein (µmol/L)b	Continuous	mixed	Baseline retinol-binding protein, trimester, month of survey, religion	Settlement
Pregnant women	Serum zinc (µg/L)	Continuous	mixed	Trimester, month of survey, religion	Settlement
Pregnant women	Mean alpha-1-acid glycoprotein (g/L)	Continuous	mixed	Baseline alpha-1-acid glycoprotein, month of survey, religion	Settlement
Pregnant women	Mean C-reactive protein (mg/L)	Continuous	mixed	Baseline C-reactive protein, month of survey, religion	Settlement
Pregnant women	Anemia[c]	Binary	melogit	Baseline hemoglobin, month of survey, religion	Settlement
Pregnant women	Iron deficiency (TBI < 0 mg/kg)[a]	Binary	melogit	Baseline total body iron value, trimester, month of survey, religion	Settlement
Pregnant women	Vitamin A deficiency (RBP < 0.7 µmol/L)[b]	Binary	melogit	Baseline retinol-binding protein, trimester, month of survey, religion	Settlement
Pregnant women	Zinc deficiency[d]	Binary	melogit	Trimester, month of survey, religion	Settlement
Pregnant women	Inflammation (ref: Healthy)[e]	Binary	melogit	Baseline alpha-1-acid glycoprotein and C-reactive protein values, trimester, month of survey, religion	Settlement
Children	Hemoglobin (g/dL)	Continuous	mixed	Average baseline hemoglobin value among children at the settlement level, child age in days as linear and quadratic variable, sex of child, month of survey, religion	Settlement, household
Children	Total body iron (mg/kg)[a]	Continuous	mixed	Average baseline total body iron value among children at the settlement level, child age in days as linear and quadratic variable, sex of child, month of survey, religion	Settlement, household
Children	Retinol-binding protein (µmol/L)[b]	Continuous	mixed	Average baseline retinol-binding protein value among children at the settlement level, child age in days as linear and quadratic variable, sex of child, month of survey, religion	Settlement, household
Children	Serum zinc (µg/L)	Continuous	mixed	Child age in days as linear and quadratic variable, sex of child, month of survey, religion	Settlement, household
Children	Mean alpha-1-acid glycoprotein (g/L)	Continuous	mixed	Average baseline alpha-1-acid glycoprotein value among children at the settlement level, child age in days as linear and quadratic variable, sex of child, month of survey, religion	Settlement, household
Children	Mean C-reactive protein (mg/L)	Continuous	mixed	Average baseline C-reactive protein value among children at the settlement level, child age in days as linear and quadratic variable, sex of child, month of survey, religion	Settlement, household
Children	Anemia[c]	Binary	melogit	Average baseline proportion of anemia among children at the settlement level, child age in days as linear and quadratic variable, sex of child, month of survey, religion	Settlement, household
Children	Iron deficiency (TBI < 0 mg/kg)[a]	Binary	melogit	Average baseline proportion of iron deficiency among children at the settlement level, child age in days as linear and quadratic variable, sex of child, month of survey, religion	Settlement, household
Children	Vitamin A deficiency (RBP < 0.7 µmol/L)[b]	Binary	melogit	Average baseline proportion of vitamin A deficiency among children at the settlement level, child age in days as linear and quadratic variable; sex of child	Settlement, household
Children	Zinc deficiency[d]	Binary	melogit	Child age in days as linear and quadratic variable, sex of child, month of survey, religion	Settlement, household
Children	Inflammation (ref: Healthy)[e]	Binary	melogit	Average baseline proportion of any inflammation among children at the settlement level, child age in days as linear and quadratic variable, sex of child, month of survey, religion	Settlement, household

Among women, baseline adjustment was done with individual blood values if available and same pregnancy status at baseline. If not available, adjustment was done with the average value of non-pregnant / pregnant women in the women's cluster. For 22 clusters, iron/vitamin A/inflammation values were not obtained for pregnant women. In these clusters, the overall mean of pregnant women at baseline was used as a baseline adjustment.

[a]Iron status was adjusted for inflammation by internal regression coefficients

[b]Vitamin A status for children was adjusted for inflammation by internal regression coefficients

[c]Anemia cut-offs: Hb < 12.0 g/dL for non-pregnant women, Hb < 11.0 g/dL for pregnant women in the first and third trimester, Hb < 10.5 g/dL for pregnant women in the second trimester, Hb < 10.5 g/dL for 6-23-month-old children, and Hb < 11.0 g/dL for ≥24-month-old children.

[d]Zinc deficiency cut-offs for women in the morning in a fasted state < 700 µg/L; morning, non-fasted: < 660 µg/L; afternoon, non-fasted: < 590 µg/L; and for children in the morning, non-fasted: < 650 µg/L; afternoon, non-fasted: < 570 µg/L. Zinc status for children was adjusted for inflammation by internal regression correction

[e]Women and children were considered to have any inflammation if C-reactive protein > 5mg/L or alpha-1-acid glycoprotein > 1g/L

Supplemental Table 5: Baseline characteristics of women and children with any baseline blood measures in the FAARM trial in Sylhet, Bangladesh

		Non-pregnant women			Pregnant women			Baseline children	
		Control	Intervention		Control	Intervention		Control	Intervention
	n	freq (%)	freq (%)	n	freq (%)	freq (%)	n	freq (%)	freq (%)
Woman's age, years	2184			349			1160		
15-19		112 (10.3)	90 (8.2)		35 (19.0)	33 (20.0)		42 (7.2)	39 (6.8)
20-24		403 (37.2)	431 (39.2)		86 (46.7)	73 (44.2)		270 (46.4)	274 (47.4)
25-29		394 (36.4)	408 (37.1)		50 (27.2)	49 (29.7)		206 (35.4)	208 (36.0)
≥ 30		175 (16.1)	171 (15.6)		13 (7.1)	10 (6.1)		64 (11.0)	57 (9.9)
Child's age, months							1160		
6-11		-	-		-	-		133 (22.9)	108 (18.7)
12-23		-	-		-	-		236 (40.6)	255 (44.1)
23-37		-	-		-	-		213 (36.6)	215 (37.2)
Woman's education	2183			349			1160		
None		173 (16.0)	179 (16.3)		26 (14.1)	23 (13.9)		95 (16.3)	23 (13.9)
Any primary		513 (47.4)	489 (44.5)		87 (47.3)	73 (44.2)		274 (47.1)	73 (44.2)
Any secondary or higher		397 (36.7)	432 (39.3)		71 (38.6)	69 (41.8)		213 (36.6)	69 (41.8)
Household wealth quintile	2183			349			1160		
First (poorest)		304 (28.1)	255 (23.2)		43 (23.4)	33 (20.0)		163 (28.0)	127 (22.0)
Second		256 (23.6)	226 (20.6)		43 (23.4)	30 (18.2)		142 (24.4)	124 (21.5)
Third		209 (19.3)	203 (18.5)		35 (19.0)	48 (29.1)		112 (19.2)	117 (20.2)
Fourth		158 (14.6)	229 (20.8)		37 (20.1)	27 (16.4)		84 (14.4)	112 (19.4)
Fifth (richest)		156 (14.4)	187 (17.0)		26 (14.1)	27 (16.4)		81 (13.9)	98 (17.0)
Religion	2184			349			1160		
Muslim		707 (65.2)	767 (69.7)		136 (73.9)	126 (76.4)		365 (62.7)	411 (71.1)
Hindu		377 (34.8)	333 (30.3)		48 (26.1)	39 (23.6)		217 (37.3)	167 (28.9)
Anemia[a]	2184			349			1160		
None		707 (65.2)	752 (68.4)		124 (67.4)	120 (72.7)		336 (57.7)	349 (60.4)
Mild		252 (23.3)	243 (22.1)		42 (22.8)	29 (17.6)		154 (26.5)	147 (25.4)
Moderate		121 (11.2)	104 (9.5)		18 (9.8)	15 (9.1)		86 (14.8)	81 (14.0)
Severe		4 (0.4)	1 (0.1)		0 (0.0)	1 (0.6)		6 (1.0)	1 (0.2)
Iron status[b]	773			133			400		
Deficient (TBI <0 mg/kg)		13 (3.4)	3 (0.8)		3 (5.0)	1 (1.4)		43 (22.1)	30 (14.6)
Deficient (SF < 15 µg/L)[c]		23 (6.0)	12 (3.1)		4 (6.7)	7 (9.6)		29 (14.9)	19 (9.3)
Deficient (sTfR < 8.3 mg/L)		12 (3.1)	3 (0.8)		3 (5.0)	3 (4.1)		66 (33.9)	65 (31.7)
Vitamin A status[d]	773			133			400		
Sufficient (RBP >1.05 µmol/L)		272 (70.7)	296 (76.3)		36 (60.0)	47 (64.4)		175 (89.7)	179 (87.3)
Insufficient (RBP 0.7-1.05µmol/L)		93 (24.2)	81 (20.9)		22 (36.7)	22 (30.1)		-	-
Deficient (RBP <0.7µmol/L)		20 (5.2)	11 (2.8)		2 (3.3)	4 (5.5)		20 (10.3)	26 (12.7)
Inflammatory markers[e]	773			133			400		
Reference		336 (87.3)	337 (86.9)		52 (86.7)	64 (87.7)		125 (64.1)	130 (63.4)
Incubation		16 (4.2)	20 (5.2)		7 (11.7)	5 (6.9)		4 (2.1)	9 (4.4)
Early convalescence		13 (3.4)	13 (3.4)		0 (0.0)	2 (2.7)		30 (15.4)	19 (9.3)
Late convalescence		20 (5.2)	18 (4.6)		1 (1.7)	2 (2.7)		36 (18.5)	47 (22.9)

FAARM: Food and Agricultural Approaches to Reducing Malnutrition; TBI: total body iron; SF: serum ferritin; sTfR: soluble transferrin receptor; RBP: retinol-binding protein; CRP: C-reactive protein; AGP: alpha-1-acid glycoprotein

Baseline characteristics are provided for women who were / were not pregnant at baseline and provided a blood measure / whose child provided a blood measure at baseline. Characteristics are for the woman / child's mother and her household. Child ages are at baseline blood measurement.

[a]Anemia cut-offs: non-pregnant women - mild: Hb 11-11.9 g/dL; moderate: 8.0-10.9 g/dL; severe: Hb < 8.0 g/dL; pregnant women in the first and third trimester - mild: Hb 10-10.9 g/dL; moderate: 7.0-9.9 g/dL; severe: Hb < 7.0 g/dL; pregnant women in the second trimester - mild: Hb 9.5-10.4 g/dL; moderate: 7.0-9.9 g/dL; severe: Hb < 7.0 g/dL; 6-23-month-old children - mild: Hb 9.5-10.4 g/dL; moderate: 7.0-9.4 g/dL; severe: Hb < 7.0 g/dL, and ≥24-month-old children - mild: Hb 10-10.9 g/dL; moderate: 7.0-9.9 g/dL; severe: Hb < 7.0 g/dL.

[b]Iron status was adjusted for inflammation by internal regression correction

[c]Different SF cut-offs used for women (< 15µg/L) and children (< 12µg/L)

[d]Vitamin A status for children was adjusted for inflammation by internal regression correction. Insufficient vitamin A status is not a

[e]Inflammatory marker categories: Incubation (CRP > 5 mg/L & AGP ≤ 1 g/L), Early convalescence (CRP > 5 mg/L & AGP > 1 g/L), Late convalescence (CRP ≤ 5 mg/L & AGP > 1 g/L)

Supplemental Table 6: Baseline and endline mean values and prevalence of anemia, micronutrient deficiencies, and inflammation among women and children enrolled in the FAARM trial in Sylhet, Bangladesh

	Non-pregnant women						Pregnant women						Children					
	Baseline			Endline			Baseline			Endline			Baseline			Endline		
	n	% or mean	(95% CI)	n	% or mean	(95% CI)	n	% or mean	(95% CI)	n	% or mean	(95% CI)	n	% or mean	(95% CI)	n	% or mean	(95% CI)
Mean hemoglobin (g/dL)	2184	12.4	(12.3, 12.5)	2278	12.6	(12.6, 12.7)	349	11.3	(11.2, 11.5)	204	11.2	(11.1, 11.4)	1160	10.9	(10.8, 11.0)	911	11.6	(11.5, 11.7)
Mean TBI (mg/kg)[a]	773	7	(6.8, 7.3)	2279	7.1	(6.9, 7.3)	133	5.9	(5.4, 6.5)	203	4.3	(3.8, 4.9)	400	2.9	(2.5, 3.3)	911	2.6	(2.2, 2.9)
Mean SF (µg/L)[a]	773	53.5	(51.1, 56.0)	2279	55.3	(53.3, 57.3)	133	48.8	(43.4, 54.3)	203	36.5	(31.4, 41.7)	400	32.8	(30.6, 35.0)	911	27.4	(25.8, 29.0)
Mean sTfR (mg/L)[a]	773	4.7	(4.5, 4.8)	2279	4.4	(4.3, 4.4)	133	5.2	(4.5, 5.9)	203	4.8	(4.5, 5.1)	400	8.6	(8.1, 9.1)	911	7.3	(7.1, 7.6)
Mean RBP (µmol/L)[b]	773	1.4	(1.4, 1.5)	2279	1.6	(1.6, 1.7)	133	1.2	(1.1, 1.2)	203	1.2	(1.1, 1.2)	400	1	(1.0, 1.0)	911	0.9	(0.9, 0.9)
Mean AGP (g/L)	773	0.6	(0.6, 0.6)	2279	0.6	(0.6, 0.6)	133	0.5	(0.4, 0.5)	203	0.4	(0.4, 0.4)	400	1	(0.9, 1.0)	911	0.9	(0.8, 0.9)
Mean CRP (mg/L)	773	1.9	(1.5, 2.3)	2279	2.3	(2.1, 2.5)	133	2.4	(1.7, 3.2)	203	3.2	(2.3, 4.1)	400	3.2	(2.5, 3.8)	911	3	(2.5, 3.5)
Anemia[c]	2184	33%	(29.9, 36.5)	2278	20%	(17.9, 22.4)	349	30%	(25.3, 34.8)	204	35%	(27.7, 42.0)	1160	41%	(37.0, 44.8)	911	16%	(13.5, 19.2)
Iron deficiency[a]																		
Deficient (TBI < 0 mg/kg)	773	2%	(0.8, 3.3)	2279	3%	(2.1, 3.7)	133	3%	(0.2, 5.9)	203	12%	(7.6, 17.0)	400	18%	(14.0, 22.5)	911	20%	(16.3, 23)
Deficient (SF < 15 µg/L)[d]	773	5%	(2.9, 6.6)	2279	7%	(5.6, 8.4)	133	6%	(2.1, 10.0)	203	31%	(24.1, 37.9)	400	12%	(8.6, 15.4)	911	21%	(17.4, 24.5)
Deficient (sTfR > 8.3 mg/L)	773	2%	(0.9, 3.0)	2279	2%	(1.5, 2.7)	133	5%	(1.1, 7.9)	203	3%	(1.0, 5.9)	400	33%	(28, 37.5)	911	20%	(17.1, 23.3)
Vitamin A deficiency[b]	773	4%	(2.5, 5.5)	2279	1%	(0.9, 1.9)	133	5%	(0.5, 8.5)	203	4%	(1.7, 7.2)	400	12%	(8.0, 15.0)	911	5%	(3.7, 6.6)
Inflammatory markers[e]																		
Reference	773	87%	(84.4, 89.3)	2279	85%	(83.5, 86.4)	133	87%	(80.5, 91.9)	203	82%	(76.3, 87.0)	400	64%	(58.9, 68.3)	911	70%	(66.3, 72.7)
Incubation	773	5%	(3.3, 6.5)	2279	7%	(5.6, 8.0)	133	9%	(5.0, 15.7)	203	14%	(10.1, 19.9)	400	3%	(1.9, 5.4)	911	2%	(1.6, 3.7)
Early convalescence	773	3%	(2.3, 4.8)	2279	4%	(3.0, 4.4)	133	2%	(0.4, 5.9)	203	0.50%	(0.1, 3.5)	400	12%	(9.7, 15.4)	911	11%	(9.6, 13.6)
Late convalescence	773	5%	(3.6, 6.7)	2279	5%	(3.9, 5.7)	133	2%	(0.7, 6.7)	203	3%	(1.4, 6.3)	400	21%	(16.5, 25.7)	911	17%	(14.2, 19.3)

FAARM: Food and Agricultural Approaches to Reducing Malnutrition; CI: confidence interval; Hb: hemoglobin; TBI: total body iron; SF: serum ferritin; sTfR: soluble transferrin receptor; RBP: retinol-binding protein; CRP: C-reactive protein; AGP: alpha-1-acid glycoprotein

[a]Iron status was adjusted for inflammation by internal regression coefficients

[b]Vitamin A status for children was adjusted for inflammation by internal regression coefficients

[c]Anemia cut-offs: Hb < 12.0 g/dL for non-pregnant women, Hb < 11.0 g/dL for pregnant women in the first and third trimester, Hb < 10.5 g/dL for pregnant women in the second trimester, Hb < 10.5 g/dL for 6-23-month-old children, and Hb < 11.0 g/dL for ≥24-month-old children.

[d]Different SF cut-offs used for women (< 15µg/L) and children (< 12µg/L)

[e]Inflammatory marker categories: Incubation (CRP > 5 mg/L & AGP ≤ 1 g/L), Early convalescence (CRP > 5 mg/L & AGP > 1 g/L), Late convalescence (CRP ≤ 5 mg/L & AGP > 1 g/L)

Supplemental Table 7: Baseline biological parameters by trimester among pregnant women in the FAARM trial in Sylhet, Bangladesh

	1st trimester		2nd trimester		3rd trimester	
	n	mean (SD) freq (%)	n	mean (SD) freq (%)	n	mean (SD) freq (%)
Mean hemoglobin (g/dL)	116	11.6 (0.1)	128	11.0 (0.1)	105	11.3 (0.1)
Mean TBI (mg/kg)[a]	48	7.0 (0.3)	45	5.5 (0.6)	40	5.2 (0.5)
Mean SF (µg/L)[a]	48	53.1 (3.7)	45	43.0 (4.2)	40	36.2 (2.8)
Mean sTfR (mg/L)[a]	48	4.6 (0.2)	45	5.9 (1.0)	40	5.2 (0.3)
Mean RBP (µmol/L)[b]	48	1.0 (0.03)	45	1.2 (0.1)	40	1.3 (0.1)
Mean AGP (g/L)	48	0.7 (0.1)	45	0.4 (0.02)	40	0.4 (0.02)
Mean CRP (mg/L)	48	2.4 (0.8)	45	1.7 (0.4)	40	3.3 (0.7)
Anemia[c]	116	33 (28.5)	128	35 (27.3)	105	37 (35.2)
Iron status[a]	48		45		40	
Deficient (TBI < 0 mg/kg)		0 (0.0)		2 (4.4)		2 (5.0)
Deficient (SF < 15 µg/L)[d]		2 (4.2)		5 (11.1)		4 (10.0)
Deficient (sTfR < 8.3 mg/L)		0 (0.0)		4 (8.9)		2 (5.0)
Vitamin A status[b]	48		45		40	
Sufficient (RBP > 1.05 µmol/L)		25 (52.1)		30 (66.7)		28 (70.0)
Insufficient (RBP 0.7-1.05µmol/L)		18 (37.5)		15 (33.3)		11 (27.5)
Deficient (RBP < 0.7 µmol/L)		5 (10.4)		0 (0.0)		1 (2.5)
Inflammatory markers[e]	48		45		40	
Reference		41 (85.4)		42 (93.3)		33 (82.5)
Incubation		2 (4.2)		3 (6.7)		7 (17.5)
Early convalescence		2 (4.2)		0 (0.0)		0 (0.0)
Late convalescence		3 (6.3)		0 (0.0)		0 (0.0)

FAARM: Food and Agricultural Approaches to Reducing Malnutrition Hb: hemoglobin; TBI: total body iron; SF: serum ferritin; sTfR: soluble transferrin receptor; RBP: retinol-binding protein; CRP: C-reactive protein; AGP: alpha-1-acid glycoprotein

[a]Iron status was adjusted for inflammation by internal regression coefficients

[b]Vitamin A status for children was adjusted for inflammation by internal regression coefficients

[c]Anemia cut-offs: 1st and 3rd trimester: < 11 g/dL; 2nd trimester: < 10.5 g/dL

[d]Different SF cut-offs used for women (< 15µg/L) and children (< 12µg/L)

[e]Inflammatory marker categories: Incubation (CRP > 5 mg/L & AGP ≤ 1 g/L), Early convalescence (CRP > 5 mg/L & AGP > 1 g/L), Late convalescence (CRP ≤ 5 mg/L & AGP > 1 g/L)

Supplemental Table 8: Endline biological parameters by trimester among pregnant women in the FAARM trial in Sylhet, Bangladesh

	1st trimester		2nd trimester		3rd trimester	
	n	mean (SD) freq (%)	n	mean (SD) freq (%)	n	mean (SD) freq (%)
Mean hemoglobin (g/dL)	36	12.0 (0.2)	65	11.0 (0.1)	103	11.1 (0.1)
Mean TBI (mg/kg)[a]	36	6.2 (0.6)	64	5.4 (0.5)	103	3.1 (0.3)
Mean SF (μg/L)[a]	36	42.7 (4.7)	64	38.9 (4.1)	103	23.5 (2.0)
Mean sTfR (mg/L)[a]	36	3.9 (0.1)	64	4.4 (0.2)	103	5.3 (0.2)
Mean RBP (μmol/L)[b]	36	1.2 (0.04)	64	1.2 (0.04)	103	1.2 (0.04)
Mean serum zinc (μg/L)	17	562.6 (27.07)	24	457.7 (19.1)	37	465.3 (16.7)
Mean AGP (g/L)	36	0.5 (0.04)	64	0.4 (0.02)	103	0.4 (0.02)
Mean CRP (mg/L)	36	1.9 (0.7)	64	4.6 (1.0)	103	2.8 (0.4)
Anemia[c]	36	3 (8.3)	65	16 (24.6)	103	52 (50.5)
Iron status[a]	36		64		103	
Deficient (TBI < 0 mg/kg)		1 (2.8)		6 (9.4)		18 (17.5)
Deficient (SF < 15 μg/L)[d]		6 (16.7)		15 (23.4)		43 (41.8)
Deficient (sTfR < 8.3 mg/L)		0 (0.0)		2 (3.1)		5 (4.9)
Vitamin A status[b]	36		64		103	
Sufficient (RBP >1.05 μmol/L)		24 (66.7)		38 (59.4)		63 (61.2)
Insufficient (RBP 0.7-1.05μmol/L)		11 (30.6)		23 (35.9)		35 (34.0)
Deficient (RBP < 0.7 μmol/L)		1 (2.8)		3 (4.7)		5 (4.9)
Zinc status[e]	17		24		37	
Deficient		9 (52.9)		18 (75.0)		27 (73.0)
Inflammatory markers[f]	36		64		103	
Reference		32 (88.9)		49 (76.6)		86 (83.5)
Incubation		1 (2.8)		13 (20.3)		15 (14.6)
Early convalescence		1 (2.8)		0 (0.0)		0 (0.0)
Late convalescence		2 (5.6)		2 (3.1)		2 (1.9)

FAARM: Food and Agricultural Approaches to Reducing Malnutrition Hb: hemoglobin; TBI: total body iron; SF: serum ferritin; sTfR: soluble transferrin receptor; RBP: retinol-binding protein; CRP: C-reactive protein; AGP: alpha-1-acid glycoprotein

[a]Iron status was adjusted for inflammation by internal regression coefficients

[b]Vitamin A status for children was adjusted for inflammation by internal regression coefficients

[c]Anemia cut-offs used were the following: 1st and 3rd trimester: < 11 g/dL; 2nd trimester: < 10.5 g/dL

[d]Different SF cut-offs used for women (< 15μg/L) and children (< 12μg/L)

[e]Zinc deficiency cut-offs for women in the morning in a fasted state < 700 μg/L; morning, non-fasted: < 660 μg/L; afternoon, non-fasted: < 590 μg/L; and for children in the morning, non-fasted: < 650 μg/L; afternoon, non-fasted: < 570 μg/L. Zinc status for children was adjusted for inflammation by internal regression correction

[f]Inflammatory marker categories: Incubation (CRP > 5 mg/L & AGP ≤ 1 g/L), Early convalescence (CRP > 5 mg/L & AGP > 1 g/L), Late convalescence (CRP ≤ 5 mg/L & AGP > 1 g/L)

Supplemental Table 9: Intra-cluster correlation coefficients and cluster sizes for anemia, micronutrient deficiencies, and inflammation among women and children enrolled in the FAARM trial in Sylhet, Bangladesh

	Non-pregnant women						Children					
	Observations per group		ICC (null model)		ICC (full model)		Observations per group		ICC (null model, 2-level)		ICC (full model, 3-level)	
	Mean	Coefficient of variation	coef.	95% CI	coef.	95% CI	Mean	Coefficient of variation	coef.	95% CI	coef.	95% CI
Hemoglobin (g/dL)												
cluster	23.7	0.43	0.04	(0.02, 0.07)	0.01	(0.00, 0.04)	9.5	0.50	0.04	(0.01, 0.10)	0.05	(0.02, 0.12)
household							1.1	0.25			0.18	(0.03, 0.60)
Total body iron (mg/kg)[a]												
cluster	23.7	0.43	0.05	(0.03, 0.08)	0.03	(0.02, 0.06)	9.5	0.49	0.07	(0.03, 0.13)	0.06	(0.03, 0.13)
household							1.1	0.25			0.33	(0.16, 0.55)
Retinol-binding protein (µmol/L)[b]												
cluster	23.7	0.43	0.02	(0.01, 0.04)	0.01	(0.004, 0.04)	9.5	0.49	0	(0.00, 0.00)	0	(0.00, 0.00)
household							1.1	0.25			0.14	(0.03, 0.45)
Serum zinc (µg/L)												
cluster	10.0	0.17	0.12	(0.07, 0.18)	0.11	(0.06, 0.17)	9.2	0.50	0.03	(0.01, 0.12)	0.02	(0.00, 0.15)
household							1.1	0.25			0.17	(0.06, 0.40)
AGP (g/L)												
cluster	23.7	0.43	0.04	(0.02, 0.07)	0.01	(0.00, 0.05)	9.5	0.49	0.01	(0.00, 0.17)	0.01	(0.00, 0.41)
household							1.1	0.25			0.01	(0.00, 0.41)
CRP (mg/L)												
cluster	23.7	0.43	0.04	(0.02, 0.07)	0.04	(0.03, 0.08)	9.5	0.49	0.00	(0.00, 0.00)	0	(0.00, 0.00)
household							1.1	0.25			0.16	(0.02, 0.62)
Anemia[c]												
cluster	23.7	0.43	0.06	(0.03, 0.12)	0	(0.00, 0.83)	9.5	0.49	0.06	(0.02, 0.20)	0.03	(0.00, 0.26)
household							1.1	0.25			0.03	(0.00, 0.26)
Iron deficiency (TBI < 0 mg/kg)[a]												
cluster	23.7	0.43	0.10	(0.03, 0.31)	0.05	(0.01, 0.37)	9.5	0.50	0.08	(0.03, 0.20)	0.03	(0.00, 0.26)
household							1.1	0.25			0.03	(0.00, 0.26)
Vitamin A deficiency (RBP < 0.7 µmol/L)[b]												
cluster	23.7	0.43	0.03	(0.00, 1.00)	0	(0.00, 1.00)	9.5	0.50	0.00	(0.00, 1.00)	0	(0.00, 1.00)
household							1.1	0.25			0	(0.00, 1.00)
Zinc deficiency[d]												
cluster	10.0	0.17	0.15	(0.09, 0.24)	0.14	(0.08, 0.24)	9.2	0.50	0.05	(0.01, 0.17)	0.05	(0.01, 0.20)
household							1.1	0.25			0.29	(0.05, 0.76)
Inflammation (ref: below cutoffs)[e]												
cluster	23.7	0.43	0	(0.00, 1.00)	0	(0.00, 1.00)	9.5	0.50	0.02	(0.00, 0.25)	0.01	(0.00, 0.47)
household							1.1	0.25			0.11	(0.00, 0.85)

FAARM: Food and Agricultural Approaches to Reducing Malnutrition; SE: standard error; Hb: hemoglobin; TBI: total body iron; RBP: retinol-binding protein; CRP: C-reactive protein; AGP: alpha-1-acid glycoprotein

Regressions were done in Stata using the commands *mixed* and *melogit,* adjusting for covariates as shown in Supplemental Table 3, followed by *estat icc* to compute intra-cluster correlation (ICC). ICC could not be estimated for inflammation among non-pregnant women and for RBP and vitamin A deficiency among children.

[a]Iron status was adjusted for inflammation by internal regression correction

[b]Vitamin A status for children was adjusted for inflammation by internal regression correction

[c]Anemia cut-offs: Hb < 12.0 g/dL for non-pregnant women, Hb < 10.5 g/dL for 6-23-month-old children, and Hb < 11.0 g/dL for ≥24-month-old children.

[d]Zinc deficiency cut-offs for women in the morning in a fasted state < 700 µg/L; morning, non-fasted: < 660 µg/L; afternoon, non-fasted: < 590 µg/L; and for children in the morning, non-fasted: < 650 µg/L; afternoon, non-fasted: < 570 µg/L. Zinc status for children was adjusted for inflammation by internal regression correction

[e]Women and children were considered to have any inflammation if CRP > 5mg/L or AGP > 1g/L.

Supplemental Table 10: Effect of the intervention and of groundwater iron levels on anemia and iron status among women and children enrolled in the FAARM trial in Sylhet, Bangladesh

	Non-pregnant women				Pregnant women				Children			
	n	OR / *beta*	95% CI	p-value	n	OR / *beta*	95% CI	p-value	n	OR / *beta*	95% CI	p-value
Hemoglobin (g/dL)	2245				202				900			
Intervention		*-0.05*	(0.85, 1.34)	0.22		*0.12*	(-0.14, 0.39)	0.37		*-0.02*	(-0.17, 0.12)	0.77
Groundwater iron quintiles (ref: lowest)												
2nd		*0.11*	(-0.01, 0.22)	0.08		*0.21*	(-0.26, 0.68)	0.38		*-0.03*	(-0.23, 0.18)	0.8
3rd (middle)		*0.16*	(0.05, 0.28)	0.007		*-0.03*	(-0.47, 0.41)	0.88		*0.006*	(-0.20, 0.21)	0.95
4th		*0.2*	(0.08, 0.32)	0.001		*0.24*	(-0.18, 0.66)	0.27		*0.29*	(0.08, 0.49)	0.01
5th (highest)		*0.2*	(0.08, 0.32)	0.001		*0.09*	(-0.33, 0.51)	0.67		*0.23*	(0.03, 0.43)	0.03
Total body iron (mg/kg)[a]	2246				201				899			
Intervention		*-0.15*	(-0.43, 0.13)	0.29		*0.38*	(-0.63, 1.38)	0.46		*0.03*	(-0.49, 0.55)	0.90
Groundwater iron quintiles (ref: lowest)												
2nd		*0.21*	(-0.18, 0.59)	0.29		*-0.8*	(-2.52, 0.92)	0.36		*-0.12*	(-0.84, 0.60)	0.75
3rd (middle)		*0.62*	(0.23, 1.00)	0.002		*-0.13*	(-1.73, 1.46)	0.87		*0.68*	(-0.04, 1.40)	0.07
4th		*0.83*	(0.44, 1.22)	<0.001		*0.9*	(-0.63, 2.44)	0.25		*1.35*	(0.63, 2.07)	<0.001
5th (highest)		*1.44*	(1.05, 1.84)	<0.001		*1.24*	(-0.28, 2.76)	0.11		*1.82*	(1.10, 2.55)	<0.001
Anemia[b]	2245				202				900			
Intervention		1.07	(0.85, 1.34)	0.58		0.61	(0.32, 1.17)	0.14		1	(0.67, 1.48)	0.99
Groundwater iron quintiles (ref: lowest)												
2nd		0.95	(0.68, 1.32)	0.76		0.78	(0.26, 2.32)	0.65		0.78	(0.44, 1.37)	0.39
3rd (middle)		0.79	(0.56, 1.11)	0.18		1.00	(0.35, 2.86)	1.00		0.61	(0.34, 1.09)	0.10
4th		0.74	(0.53, 1.05)	0.09		0.59	(0.22, 1.61)	0.30		0.38	(0.20, 0.70)	0.002
5th (highest)		0.68	(0.47, 0.97)	0.04		0.59	(0.22, 1.57)	0.29		0.43	(0.23, 0.81)	0.01
Iron deficiency (TBI < 0 mg/kg)[a]	2246				201				899			
Intervention		0.96	(0.56, 1.65)	0.88		1.09	(0.34, 3.54)	0.88		0.84	(0.44, 1.57)	0.58
Groundwater iron quintiles (ref: lowest)												
2nd		0.7	(0.36, 1.38)	0.31		2.66	(0.52, 13.6)	0.24		1.08	(0.50, 2.33)	0.85
3rd (middle)		0.31	(0.13, 0.71)	0.006		0.69	(0.13, 3.56)	0.66		0.36	(0.14, 0.95)	0.04
4th		0.39	(0.17, 0.87)	0.02		0.42	(0.08, 2.36)	0.33		0.21	(0.06, 0.70)	0.01
5th (highest)		0.26	(0.10, 0.68)	0.006		0.07	(0.01, 0.86)	0.04		0.13	(0.03, 0.55)	0.01

FAARM: Food and Agricultural Approaches for Reducing Malnutrition; OR: odds ratio; CI: confidence interval; Hb: hemoglobin; TBI: total body iron

Groundwater iron quintiles were calculated using the full FAARM survey sample with the following iron values per quintile: 1st, 0.1-2.8mg/L; 2nd, 2.8-4.3mg/L; 3rd, 4.3-6.8mg/L; 4th, 6.8-10.8mg/L; 5th, 10.9-41.8mg/L.

Households that did not identify tubewell water as their main drinking source or were not surveyed were excluded from these analysis (non-pregnant women: n=33; pregnant women: n=2; children: (hemoglobin) n=11, (iron) n=12)

Regression models included settlement-level and household-level random effects and were adjusted for the variables shown. Child regressions were additionally adjusted for age as a linear and quadratic term.

Beta coefficients are written in italics.

[a]Iron status was adjusted for inflammation by internal regression coefficients

[b]Anemia cut-offs: Hb < 12.0 g/dL for non-pregnant women, Hb < 11.0 g/dL for pregnant women in the first and third trimester, Hb < 10.5 g/dL for pregnant women in the second trimester, Hb < 10.5 g/dL for 6-23-month-old children, and Hb < 11.0 g/dL for ≥24-month-old children.
