# Supplementary material for: Impacts of a Homestead Food Production Intervention on Anaemia and Micronutrient Deficiencies Among Women and Children in Rural Bangladesh: A Cluster‐Randomized Controlled Trial
Source: Matern Child Nutr. 2025 May 19;21(4):e70043. doi: 10.1111/mcn.70043 (PMC12454213; doi:10.1111/mcn.70043)
Supplement: Supplementary file 3 — Supporting information_texts_final. [file MCN-21-e70043-s003.pdf]

Supplemental text files

- Supplemental text 1: Study design and sampling
- Supplemental text 2: Baseline and endline blood collection
- Supplemental text 3: Water collection and laboratory analyses of groundwater samples
- Supplemental text 4: Blood sample laboratory analyses
- Supplemental text 5: Cut-offs and adjustments for anemia, iron deficiency, and zinc deficiency

Supplemental Text 1

Study design and sampling

Randomization

Randomization was done at the unit of a geographically contiguous settlement, in which there were at least 10 women eligible for recruitment into the trial. A 400-meter buffer was maintained between settlements to prevent spillover.

Blinding

Participants in the FAARM trial and data collectors were not explicitly informed about settlement allocation. Data analysts were unblinded so as to track outcomes and enable potential improvements of program implementation during the course of the trial.

Blood measurement and analysis sampling

In both the baseline and endline survey, hemoglobin concentration was measured in all available blood samples from women and children. In the baseline survey, only a sub-sample of women's and children's blood was analyzed for additional measures (i.e., iron, vitamin A, and inflammatory measures) due to financial constraints. The random sub-sample was comprised of 9 households per cluster from each of the 96 settlements for a total of 864 households (934 women and 401 children). Zinc status was not measured at baseline as very little volume was collected and zinc-free materials were not used. In the endline survey, all available blood samples were analyzed for iron and vitamin A status as well as inflammatory biomarkers. Zinc status was analyzed in all children's and a random sub-sample of women's blood samples.

Supplemental Text 2

Baseline and endline blood collection

Baseline blood collection

During the baseline survey, capillary blood (300 µL) was collected from women and their children over six months of age (≥183 days) by trained phlebotomists. Hemoglobin concentrations were measured on-site using the Hemocue 201+ System (HemoCue AB, Sweden) with the third drop of capillary blood according to standard procedures. Additional capillary blood was collected in Microvette™ tubes and transported to the field lab at the end of each day in insulated bags with ice packs to maintain a cold chain (4-8°C). Blood samples were refrigerated (~4°C) overnight, and the following morning, were centrifuged at 12,100 x g for 10 minutes. The supernatant was pipetted off into 50 µL aliquots of serum. Serum samples were stored at -20°C and transported to Germany on dry ice. Samples were then stored at -20°C until further analyses.

Endline blood collection

During the endline survey, trained phlebotomists collected venous blood from enrolled women (8 mL) and children (5 mL) from the antecubital area of the arm using standard methods. Blood was collected into two vacutainers: one containing EDTA (dipotassium ethylenediaminetetraacetic acid) (1,8 mg/mL) for hematology, and trace-element-free tubes for serum analysis (BD; Franklin Lakes, NJ, USA). Vacutainers were transported to the field lab twice daily, maintaining a cold chain (4-8°C). At the field lab, native blood samples were centrifuged at 1500 x g for 15 minutes for serum separation. Serum (100 µL) was then pipetted into a 0.5 mL sample tube (Micronic; Lelystad, The Netherlands) and, depending on available sample volume, up to 700 µL each in three Eppendorf tubes (Eppendorf; Hamburg, Germany). EDTA samples were used for complete blood count analysis using the Sysmex XP-100 (Sysmex; Kobe, Japan), including estimates of hemoglobin concentration. Serum samples were stored on-site in a -20°C freezer until transport to Germany on dry ice, where they were stored at -20°C. International transport was done through World Courier™ and temperatures were monitored during shipping. Transport within Germany to laboratories for analysis was done with dry ice. At blood collection, fasting status and time of blood sampling were noted.

Supplemental Text 3

Water collection and laboratory analyses of groundwater samples

To ensure fresh groundwater was sampled, tubewells were pumped for five minutes before collection. Water samples were filled in 15 mL tubes through a 0.2 µm membrane syringe filter to remove particulate matter. For cation measurement, tubes were acidified with 100 µL of 6 Mol hydrochloric acid to maintain a pH of less than 2. This prevented iron and other oxidizable substances from precipitation. Acidified samples were stored at room temperature until measurement.

Groundwater iron measurements were conducted at the Institute of Earth Sciences in Heidelberg with an Inductively Coupled Plasma – Optical Emission Spectroscopy (ICP-OES, ICP 720 ES, Agilent Technologies, Inc.; Santa Clara, USA) using standard methodology adapted from DIN EN ISO 11885:2009. The specific setup of the ICP-OES was with Axial Induction coil, long torch and a VistaChip II CCD-Detector (Agilent Technologies, Inc.; Santa Clara, USA). A multi-standard (ICP multi-element standard solution IV) (Merck KGaA; Darmstadt, Germany) for calibration and the reference materials SWS-SW2, SPS-WW2 (Spectrapure Standards AS, Norway) and TMDA 61.3 (Environment and Climate Change, Canada) for quality control were used. For iron, a maximum relative error of 1% for all measurements and a relative standard deviation of between 2.8% and 3.2% were determined. Iron was detected at a wavelength of 261.187 nm for high concentrations (calibration from 1,000 to 50,000 µg/L) and at a wavelength of 238.204 nm for low concentrations (calibration from 0 to 500 µg/L).

Supplemental Text 4

Blood sample laboratory analyses

Iron, vitamin A, and inflammation measures

All values are the mean of an independent double measurement. Measures with a coefficient of variation (CV) higher than 15% were repeated and obvious outliers were removed. For calibration, quality control samples from quality assurance programs of Biorad and the United States Centers for Disease Control were used with a wide range of values. Levels of detection were as follows: serum ferritin 5µg/L, soluble transferrin receptor 0.5 mg/L, retinol-binding protein 0.1µmol/L, C-reactive protein 0.5 mg/L, and alpha-1-acid glycoprotein 0.1 g/L.

Zinc measures

The mass concentrations of serum zinc were determined using a Perkin Elmer 300x mass spectrometer with inductively coupled plasma (ICP-MS). Prior to measurement, the sample solution was diluted 1:100. An internal standard, Rhodium ICP Standard 1000 mg/L Rh CertiPUR® (Merck; Lot number: OC567544), was added to ensure the accurate detection of low concentrations. For calibration, a CAL-2 standard solution and an internal standard, Instrument Calibration Standard 2 with 26 components (Honeywell; Product number E2149; Lot number: M098A), was used. A calibration line was determined (from 0.1 µg/L to 20 µg/L) and used to quantify the element concentrations in the sample solutions.

Supplemental Text 5

Cut-offs and adjustments for anemia, iron deficiency, and zinc deficiency

Anemia

Anemia was defined based on hemoglobin (Hb) concentrations according to World Health Organization recommendations for non-pregnant women (mild: Hb 11-11.9 g/dL; moderate: 8.0-10.9 g/dL; severe: Hb < 8.0 g/dL), 6-23-month-old children (mild: Hb 9.5-10.4 g/dL; moderate: 7.0-9.4 g/dL; severe: Hb < 7.0 g/dL), and 24-59-month-old children (mild: Hb 10-10.9 g/dL; moderate: 7.0-9.9 g/dL; severe: Hb < 7.0 g/dL) [1]. The WHO also recently updated their recommendations for trimester-specific anemia cut-offs during pregnancy for pregnant women in the first and third trimesters (mild: Hb 10-10.9 g/dL; moderate: 7.0-9.9 g/dL; severe: Hb < 7.0 g/dL) and second trimester (mild: Hb 9.5-10.4 g/dL; moderate: 7.0-9.4 g/dL; severe: Hb < 7.0 g/dL). No adjustments were required for smoking or elevation.

Iron deficiency

Serum ferritin (SF) values below 15 µg/L for women and below 12 µg/L for children were defined as iron deficiency after adjustment for inflammation, using the internal regression correction approach according to Biomarkers Reflecting Inflammation and Nutritional Determinants of Anemia (BRINDA) recommendations [2]. Soluble transferrin receptor (sTfR) concentrations above 8.3 mg/L were defined as iron deficiency according to VitMin laboratory standards, as to date, there are no international standards [3, 4]. Adjustment of sTfR values for inflammation was conducted following the BRINDA approach [4]. Total Body Iron (TBI) was calculated based on SF and sTfR values (TBI= -(log10(sTfR*1000)/SF) - 2.8229) / 0.1207) [5, 6]. TBI values were also adjusted for inflammation according to BRINDA-recommended procedures [7]. We used the same cut-offs to define iron deficiency throughout all pregnancy periods for SF, sTfR, and TBI. Measures of SF during pregnancy should be interpreted with caution as they can be affected by hemodilution [8]. In contrast, sTfR has been shown not to be influenced by pregnancy [9], and total body iron has also been proposed as a reliable indicator during pregnancy, although it has not been validated yet [5, 10, 11].

Zinc deficiency

Zinc deficiency was defined according to time of measurement and fasting status, following recommendations from the International Zinc Nutrition Consultative Group [12]. Among non-pregnant women, zinc deficiency was defined as < 70 µg/dL (morning, fasting), < 66 µg/dL (morning, non-fasting), < 59 µg/dL (afternoon/evening). Among pregnant women, zinc cut-offs were trimester-specific (first trimester: < 56 µg/dL; second and third trimesters: < 50 µg/dL). Only 21 non-pregnant women and 1 pregnant woman were classified as fasting

(i.e., had not eaten in the past 8 hours). As there are no universally accepted reference values for zinc deficiency for children under 3 years of age, current recommendations are to apply the standard cut-offs for children less than 10 years old: < 65 µg/L (morning, non-fasting), < 57 µg/L (afternoon/evening) [12]. Serum zinc concentrations and deficiency estimates among children were adjusted using internal regression correction using the BRINDA approach [13, 14].

References

1. World Health Organization (WHO): **Guideline on haemoglobin cutoffs to define anaemia in individuals and populations.** Geneva: WHO; 2024.
2. Namaste SM, Rohner F, Huang J, Bhushan NL, Flores-Ayala R, Kupka R, Mei Z, Rawat R, Williams AM, Raiten DJ, et al: **Adjusting ferritin concentrations for inflammation: Biomarkers Reflecting Inflammation and Nutritional Determinants of Anemia (BRINDA) project.** *Am J Clin Nutr* 2017, **106:**359s-371s.
3. Erhardt JG, Estes JE, Pfeiffer CM, Biesalski HK, Craft NE: **Combined measurement of ferritin, soluble transferrin receptor, retinol binding protein, and C-reactive protein by an inexpensive, sensitive, and simple sandwich enzyme-linked immunosorbent assay technique.** *J Nutr* 2004, **134:**3127-3132.
4. Rohner F, Namaste SM, Larson LM, Addo OY, Mei Z, Suchdev PS, Williams AM, Sakr Ashour FA, Rawat R, Raiten DJ, Northrop-Clewes CA: **Adjusting soluble transferrin receptor concentrations for inflammation: Biomarkers Reflecting Inflammation and Nutritional Determinants of Anemia (BRINDA) project.** *Am J Clin Nutr* 2017, **106:**372s-382s.
5. Cook JD, Flowers CH, Skikne BS: **The quantitative assessment of body iron.** *Blood* 2003, **101:**3359-3364.
6. Skikne BS, Flowers CH, Cook JD: **Serum transferrin receptor: a quantitative measure of tissue iron deficiency.** *Blood* 1990, **75:**1870-1876.
7. Mei Z, Namaste SM, Serdula M, Suchdev PS, Rohner F, Flores-Ayala R, Addo OY, Raiten DJ: **Adjusting total body iron for inflammation: Biomarkers Reflecting Inflammation and Nutritional Determinants of Anemia (BRINDA) project.** *Am J Clin Nutr* 2017, **106:**383s-389s.
8. Centers for Disease Control and Prevention: **Recommendations to prevent and control iron deficiency in the United States.** *MMWR* 1998, **47**.
9. **Assessing the iron status of populations: including literatures reviews.** In *Report of a Joint World Health Organization/Centers for Disease Control and Prevention Technical Consultation on the Assessment of Iron Status at the Population Level.* 2nd edition. Geneva, Switzerland: World Health Organization; 2004
10. Mei Z, Cogswell ME, Looker AC, Pfeiffer CM, Cusick SE, Lacher DA, Grummer-Strawn LM: **Assessment of iron status in US pregnant women from the National Health and Nutrition Examination Survey (NHANES), 1999–2006.** *The American Journal of Clinical Nutrition* 2011, **93:**1312-1320.
11. Iannotti LL, O'Brien KO, Chang SC, Mancini J, Schulman-Nathanson M, Liu S, Harris ZL, Witter FR: **Iron deficiency anemia and depleted body iron reserves are prevalent among pregnant African-American adolescents.** *J Nutr* 2005, **135:**2572-2577.
12. International Zinc Nutrition Consultative Group (IZiNCG): **IZiNCG technical document #1. Assessment of the risk of zinc deficiency in populations and options for its

control. (Brown KH, Rivera JA, Bhutta Z, Gibson RS, King JC, Lönnerdal B, Ruel M, Sandtröm B, Wasantwisut E, Hotz C*, et al* eds.), vol. 25. pp. S91-S204 2004:S91-S204
13. McDonald CM, Suchdev PS, Krebs NF, Hess SY, Wessells KR, Ismaily S, Rahman S, Wieringa FT, Williams AM, Brown KH, King JC: **Adjusting plasma or serum zinc concentrations for inflammation: Biomarkers Reflecting Inflammation and Nutritional Determinants of Anemia (BRINDA) project.** *Am J Clin Nutr* 2020, **111:**927-937.
14. Namaste SM, Aaron GJ, Varadhan R, Peerson JM, Suchdev PS: **Methodologic approach for the Biomarkers Reflecting Inflammation and Nutritional Determinants of Anemia (BRINDA) project.** *Am J Clin Nutr* 2017, **106:**333s-347s.
